# On Inertial Body Tracking in the Presence of Model Calibration Errors

**DOI:** 10.3390/s16071132

**Published:** 2016-07-22

**Authors:** Markus Miezal, Bertram Taetz, Gabriele Bleser

**Affiliations:** Junior Research Group wearHEALTH, University of Kaiserslautern, Gottlieb-Daimler-Str. 48, 67663 Kaiserslautern, Germany; miezal@cs.uni-kl.de (M.M.); taetz@cs.uni-kl.de (B.T.)

**Keywords:** inertial body tracking, biomechanical model, calibration, magnetometers, sensor fusion, extended Kalman filter, optimization

## Abstract

In inertial body tracking, the human body is commonly represented as a biomechanical model consisting of rigid segments with known lengths and connecting joints. The model state is then estimated via sensor fusion methods based on data from attached inertial measurement units (IMUs). This requires the relative poses of the IMUs *w.r.t.* the segments—the IMU-to-segment calibrations, subsequently called I2S calibrations—to be known. Since calibration methods based on static poses, movements and manual measurements are still the most widely used, potentially large human-induced calibration errors have to be expected. This work compares three newly developed/adapted extended Kalman filter (EKF) and optimization-based sensor fusion methods with an existing EKF-based method *w.r.t.* their segment orientation estimation accuracy in the presence of model calibration errors with and without using magnetometer information. While the existing EKF-based method uses a segment-centered kinematic chain biomechanical model and a constant angular acceleration motion model, the newly developed/adapted methods are all based on a free segments model, where each segment is represented with six degrees of freedom in the global frame. Moreover, these methods differ in the assumed motion model (constant angular acceleration, constant angular velocity, inertial data as control input), the state representation (segment-centered, IMU-centered) and the estimation method (EKF, sliding window optimization). In addition to the free segments representation, the optimization-based method also represents each IMU with six degrees of freedom in the global frame. In the evaluation on simulated and real data from a three segment model (an arm), the optimization-based method showed the smallest mean errors, standard deviations and maximum errors throughout all tests. It also showed the lowest dependency on magnetometer information and motion agility. Moreover, it was insensitive *w.r.t.* I2S position and segment length errors in the tested ranges. Errors in the I2S orientations were, however, linearly propagated into the estimated segment orientations. In the absence of magnetic disturbances, severe model calibration errors and fast motion changes, the newly developed IMU centered EKF-based method yielded comparable results with lower computational complexity.

## 1. Introduction

Inertial motion capturing has found widespread use in various applications, including biomechanics and health as two prominent ones [1,2,3,4]. This development is, among other reasons, driven by the availability of smaller, cheaper and more precise hardware [4]. Inertial measurement units (IMUs) comprise gyroscopes and accelerometers providing 3D acceleration and 3D rotational velocity. In most cases they also contain magnetometers adding 3D magnetic fields. This is also referred to as MIMUs. From these measurements motion information can be estimated through sensor fusion.

A considerable amount of literature deals with fusion techniques for orientation tracking based on a single IMU [5,6,7,8,9,10]. Especially the separation of body acceleration and acceleration due to gravity [6,8,9,11] and the handling of magnetic disturbances [6,10] received attention. Many applications, however, require knowledge about the motion of several (connected) body segments, often in terms of joint angles, as derived from multiple attached IMUs [12,13,14]. In this case, rather than independently tracking the orientation of each segment by means of the attached IMU, biomechanical models of the human body including IMU-to-segment placement are used in the sensor fusion method for reducing tracking errors [15,16,17,18,19,20]. Here, the body is typically modeled as a set of rigid segments (the bones) with known lengths, which are connected through frictionless joints of varying degrees of freedom (DoFs), and the IMUs are assumed to be rigidly mounted on the associated segments (mostly with a one-to-one mapping) [21]. The respective transformation, i.e., the orientation and position of each IMU *w.r.t.* the segment it is attached to, is called IMU-to-segment (subsequently abbreviated I2S) calibration [22].

It is obvious that such model assumptions introduce errors associated to these assumptions. In this work, we are focusing on the influence of selected model calibration errors, more specifically, on errors in the calibrated I2S orientations, positions and segment lengths, on the segment orientation estimation accuracy. Other calibration errors (e.g., joint rotation axes [23]) and errors associated to the simplification of the biomechanical model (e.g., limited joint DoFs [19] or soft tissue artifacts [24], which are motion reconstruction errors of the skeleton due to movement of e.g., skin or cloths *w.r.t.* the bone) are not in the focus here.

Sensor fusion algorithms were developed to better handle or compensate for sensor errors [8,9,20,25], such as noise and bias, and environmental effects, such as magnetic disturbances [6,10]. In contrast, model calibration errors were not intensively addressed in literature; though they represent a significant source of error [25] (cf. Section 1.2). Therefore, this work proposes three new/adapted sensor fusion methods with the aim of providing increased robustness against such model calibration errors. The performances of these methods, in combination with their dependence on magnetometer usage, are assessed in comparison to an existing method [16]. Note, the above mentioned sensor errors and compensation strategies for magnetic disturbance effects are not in the focus here. In the following, Section 1.1 reviews state-of-the-art sensor fusion methods, which provide the basis for the proposed set of methods. Section 1.2 shortly summarizes state-of-the-art methods for obtaining the addressed calibration parameters, which provides an indication of typical error ranges. The contributions of this work are then detailed in Section 1.3.

### 1.1. Sensor Fusion Methods

Based on the assessed literature, sensor fusion methods are in this work mainly distinguished through the chosen biomechanical model representation and the method for solving the resulting estimation problem. They are also categorized *w.r.t.* magnetometer usage.

**Figure 1:**
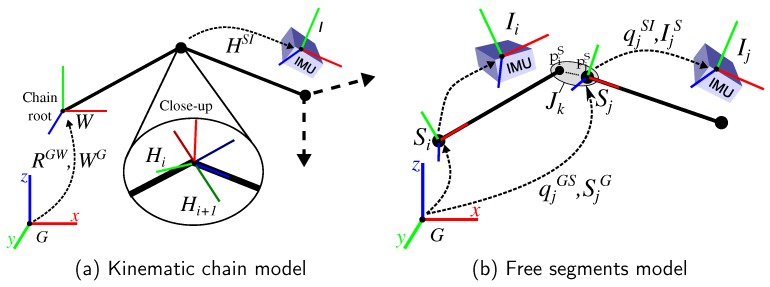
Two different biomechanical model representations. Note the additional world coordinate system in the kinematic chain model.

A widespread and efficient representation of a kinematic body model is via a kinematic chain, e.g., [13,16,18,20]. Here, the global orientation and position of the root segment, as well as the *relative* orientations between segments (cf. Figure 1a), i.e., the joint angles, are modeled as estimation variables. Orientations are typically parametrized in a minimal way, e.g., through Euler angles [13] or Denavit-Hartenberg (DH) coordinates [16,20]. *Kinematic chain models* offer several advantages: They can be used for predicting body accelerations at the IMU positions, which aids the separation from accelerations due to gravity. In case of a minimal rotation parametrization and, if the segment coordinate systems are sufficiently well aligned with the anatomical rotation axes, restricted joint DoFs can be easily modeled by omitting single angular DoFs. Moreover, relative joint angles are the variables of interest for most applications, e.g., [12,13,14]. At the same time, minimal orientation parametrizations suffer from singularities [26].

Another approach found in literature chooses a redundant parametrization for the biomechanical model by representing each segment with an orientation (mostly via a singularity-free unit quaternion [26]) and position (or velocity [27]) *w.r.t.* a global frame [17,19,28]. This is subsequently referred to as *free segments model* (cf. Figure 1b). Conditions from the biomechanical model (e.g., connected segments, restricted rotational DoFs) are then incorporated into the estimation as stochastic constraints, e.g., via virtual measurements [17,19]. In [19] this concept is also extended to the IMU placement. Here, not only global segment kinematics but also the global poses of all IMUs are estimated. The rigid connections between the IMUs and segments, i.e., the I2S calibrations, are then again incorporated via stochastic constraints. Obviously, the definition of a redundant system with stochastic constraints increases the dimensionality of the estimation problem, which results in a higher computational complexity compared to the kinematic chain parametrization (cf. Table 1). At the same time stochastic constraints consider errors in the associated biomechanical model assumptions and parameters.

Along with the biomechanical model representation there are also different methods for solving the resulting estimation problem. Focusing on online applications the most widespread methods are based on recursive filters, e.g., on an extended Kalman filter (EKF) [9,16,27]. The latter approximates nonlinear motion and measurement equations using a first-order Taylor expansion around the current estimate [29]. The unscented Kalman filter (UKF) [6,20] uses sigma-point approximations [30] that are related to the second-order moments [31]. Note that the UKF does not necessarily yield better performance than the EKF, which has been investigated for the inertial tracking of an arm in [20]. In contrast to these filtering approaches, the work of [19] proposes an optimization-based method to inertial body motion tracking. Here, an offline maximum a posteriori smoothing estimate of the segment kinematics and IMU poses is obtained from a sequence of inertial measurements by solving a global constrained weighted least squares (WLS) problem using an infeasible start Gauss-Newton method. In [32], the estimation problem is decomposed into small subproblems exploiting its sparsity structure. This leads to a distributed, but still offline version of the method, which allows for making use of multiple processors. Using an optimization formulation better accounts for nonlinearities through repeated linearizations. Moreover, it enables the incorporation of global constraints and non-Gaussian noises. Note, the EKF can be considered as a special case of one optimization step [33]. Obviously, an optimization-based estimate is, compared to a filtering approach, computationally more expensive (cf. Table 1). Moreover, the methods in [19,32] are offline, since all measurements need to be collected before processing.

As mentioned above, inertial sensors are typically combined with magnetometers in order to compensate for heading drift resulting from the integration of noisy and biased gyroscope measurements. To this end, the work of [10] provides a survey of different techniques for dealing with magnetic disturbances. From the above referenced methods [18,19,20,27,32] work magnetometer-free and can thus be considered independent of a static magnetic field.

### 1.2. Calibration Methods

In order to derive I2S orientations, calibration procedures, which require the user to precisely perform predefined static poses [16,17,34] or movements [35,36], are typically used. In a recent study [22] different established calibration methods were validated against an optical reference system based on ten healthy subjects instructed by three operators. The study reports trueness (root mean squared error *w.r.t.* the optical reference) in the range ${\left[8;26\right]}^{\circ }$ and precision (reproducibility) in the range ${\left[5;10\right]}^{\circ }$, which attests that potentially large (human-induced) errors *w.r.t.* the I2S orientations have to be expected, even when subjects are instructed when performing the calibration. Concerning the I2S positions, there are no established calibration methods. In [37], an offline least squares estimator is proposed, which obtains the position of a ball-and-socket joint in the reference frame of two IMUs attached to the adjacent segments from arbitrary motion. The calibration method is used in [18] as basis for knee flexion/extension and ankle plantar/dorsiflexion angle estimation. On simulated data from a three segment model, the precision of the position calibration method is reported with less than three percent difference to the true values. On real data from one subject, the repeatability is reported with variations by about ±0.01 m. Manually measuring the I2S positions or making assumptions concerning their relative positions *w.r.t.* the associated segment seem to be the most widespread methods [16,17,19,27,38]. Segment lengths are often also manually measured, based on anatomical landmarks, or they are derived from a measured or assumed height, based on anthropometric tables [16,17,19]. Both methods likely lead to errors in the order of several centimeters, given the difficulty in precisely locating the joint centers, even based on anatomical landmarks [39].

Note, besides the above mentioned position self-calibration method [37], Taetz et al. [40] propose an online capable system, an extension of the optimization-based approach presented in this work, where both I2S positions and orientations are estimated simultaneously with the segment poses. On simulated data from a two segment model, an average precision in the order of sub-degrees for the estimated I2S orientations and in the order of 0.01 m for the estimated I2S positions is reported. On real data from the lower body of one subject, the repeatability of the I2S orientation estimation is noted with a variation of below $2^{\circ }$ for four IMUs mounted on the upper and lower legs. The estimated I2S positions seemed to be more accurate for the upper legs (repeatability < 0.01 m) than for the lower legs (<0.06 m), which is explained by the low amount of motion variability in the lower legs. Hence, while self-calibration methods appear as a promising way for reducing human-induced calibration errors, they are subject of current research and seem to be not yet in the state of being widely used.

### 1.3. Contributions

Given that calibration methods based on static poses, movements and manual measurements are still the most widely used, potentially large calibration errors have to be expected, in particular when calibration is performed by inexperienced users. A practical inertial body tracking method should therefore robustly handle such errors. Showing reduced dependence on magnetometer usage also represents an important aspect of robustness in man made environments. See, for instance, [41] for an evaluation of indoor magnetic distortion effects on gait analysis performed with wearable inertial sensors. In this respect, the major contributions of this work are:

- The development of two EKF-based methods with different state-space models, which use the free-segments representation, inspired by [17]. These are subsequently denoted *Quattracker IMU* and *Quattracker segment*. Here, rotations are represented through unit quaternions.
- The development of an online capable version of the optimization-based method in [19], based on sliding window optimization. The method is subsequently denoted *Optitracker*.
- A performance comparison of the new/adapted methods with an existing EKF-based method that uses the kinematic chain representation and DH coordinates to represent joint angles [16]. Performance is measured in terms of angular error statistics on complex (i.e., simultaneous variations in all joint DoFs) moderate and fast human motion (real and simulated) and on artificially simulated complex motion from a case study. In particular, the influence of the selected model calibration errors, i.e., I2S calibration and segment length errors, on the performances of the different methods and their dependence on magnetometer usage are assessed.

Based on the results from these studies, useful considerations concerning the usage of the different tested methods are provided. In the following, the notation, methods and evaluation setup are introduced in Section 2. The results are summarized in Section 3. Section 4 discusses these results and draws conclusions.

## 2. Materials and Methods

### 2.1. Notation

Let A,B be two Cartesian coordinate systems (coordinate frames), then $R^{AB}\in SO\left(3\right)$ denotes the orientation of frame *B* in frame *A* and $A^{B}\in R^{3}$ denotes the translation of frame *B* in frame *A*. The rotation matrix $R^{AB}$ and its unit quaternion representation $q^{AB}$ are used interchangeable. Moreover, $H^{AB}$ denotes a homogeneous transformation comprising both, orientation and position $\left\{R^{AB},A^{B}\right\}$. To switch between the representations, $rot\left(H^{AB}\right)R^{AB}$ extracts the rotation matrix from a homogeneous transformation. The translation is extracted with $trans\left(H^{AB}\right)A^{B}$. The inverse rotation is denoted ${\left(q^{AB}\right)}^{*}q^{BA}$, ${\left(R^{AB}\right)}^{T}=R^{BA}$. The angular velocity of frame *B w.r.t. A* in frame *A* is denoted $\omega_{A}^{AB}$. The cross product v×w with $v,w\in R^{3}$ can be rewritten as a matrix multiplication S(v)w, with S(v) being a skew-symmetric matrix. Let $a_{t}\in R^{m}$ be a time dependent variable of dimension *m*, then ${\dot{a}}_{t}$ denotes the first and ${\ddot{a}}_{t}$ the second time derivative. Process and measurement noises are assumed to be additive and Gaussian distributed with zero mean. Measurement noises are denoted $e_{t}^{X}∼N\left(0,\Sigma^{X}\right)$, with $\Sigma^{X}$ being the noise covariance matrix, while process noises are denoted $e_{t}^{\hat{X}}∼N\left(0,\Sigma^{\hat{X}}\right)$. Acceleration, angular velocity and magnetic field measurements from IMU *i* at time *t* are denoted $y_{i,t}^{a},y_{i,t}^{\omega },y_{i,t}^{m}\in R^{3}$, respectively.

### 2.2. Biomechanical Model Representations

The two biomechanical model representations, i.e., the *kinematic chain model* and the *free segments model* (see Figure 1), are now formalized using the introduced notation. Both representations share a global position-less coordinate system *G*, whose *x*-axis is aligned with the local magnetic north and whose *z*-axis points up, opposite gravity. Each segment *i* has a local coordinate frame $S_{i}$ and is assumed to have one IMU attached to it. Thus, for notational brevity, segment and attached IMU share one index. Each IMU *i* has a local coordinate frame $I_{i}$, in which the measurements are represented. Moreover, $S_{i}$ and $I_{i}$ are rigidly connected via the I2S orientation $q_{i}^{SI}$ and position $I_{i}^{S}$ (or $H_{i}^{SI}$ for the chain model). The kinematic chain model, illustrated in Figure 1a, defines a hierarchical order of transformations. In this work, the definitions from [16] are used, which are based on DH transformations [42]. For the sake of completeness, the transformations and equations for the model construction are provided in Appendix A. Further details can be found in [16]. The free segments model is composed of a set of segments $S_{i}\in S$, a set of IMUs $I_{i}\in I$ and a set of joints $J_{k}\in J$. For each intermediate joint $J_{k}$, the set of connected segments is denoted $S_{i},S_{j}\in S_{J_{k}}$. Each segment $S_{i}$ and IMU $I_{i}$ is represented in *G* with an orientation and translation {$q_{i}^{GS}$, $S_{i}^{G}$} and {$q_{i}^{GI}$, $I_{i}^{G}$}, respectively. Figure 1b illustrates this model. Note, each segment also has a start and an endpoint defined in the local segment coordinate system ($p_{i}^{S}$, $p_{j}^{S}$ in the figure), which are used in Equation (10).

### 2.3. EKF-Based Methods

The *Chaintracker*, the *Quattracker IMU* and the *Quattracker segment* use an EKF for parameter estimation. The former is taken from [16] and is enhanced with an initialization method described in Section 2.3.5. The other methods are inspired by the work of [17,19]. Note, the work of [17] provides only a general concept for combining a free segments model with biomechanical constraints. The formalizations with different state-space models leading to the *Quattracker IMU* and *Quattracker segment* were contributed as part of this manuscript.

#### 2.3.1. Measurement Models

For IMU $I_{i}$, the accelerometer measurement model at time *t* is:

(1) $$y_{i,t}^{a}={\left(R_{i,t}^{GI}\right)}^{T}\left({\ddot{I}}_{i}^{G}-g^{G}\right)+e_{i,t}^{a}$$

Here, ${\ddot{I}}_{i}^{G}$ and $g^{G}$ denote body acceleration and acceleration due to gravity, respectively, both given in the global frame.

The gyroscopes measure the angular velocities *w.r.t.* the global frame, transformed into the IMU frame, resulting in the following gyroscope measurement model:

(2) $$\begin{array}{ccc} y_{i,t}^{\omega } & = & \omega_{I,i,t}^{GI}+e_{i,t}^{\omega } \end{array}$$

Note, gyroscope and accelerometer bias models are not in the focus of this paper, but can be easily added, see e.g., [19,43].

The magnetometer measurement model was chosen to only have an effect on the estimated yaw direction. It omits information concerning the local dip angle. This is a common way of reducing the influence of magnetic disturbances [10]. Let ${\hat{y}}_{i,t}^{m}$ be the normalized magnetometer measurement, then the model can be written as:

(3) $$0=\mathrm{atan}2\left[\frac{{\left(R_{i,t}^{GI}\;{\hat{y}}_{i,t}^{m}\right)}_{y}}{{\left(R_{i,t}^{GI}\;{\hat{y}}_{i,t}^{m}\right)}_{x}}\right]+e_{i,t}^{m}$$

Here, ${\left(\;\cdot \;\right)}_{x}$, ${\left(\;\cdot \;\right)}_{y}$ denote the *x*- and *y*-component of the vector. Hence, the model considers the angular deviation of the magnetometer measurement, as transformed into the global frame and projected into the horizontal plane, from the global *x*-axis.

In all models, the three variables ${\ddot{I}}^{G}$, $\omega_{I}^{GI}$ and $R^{GI}$ need to be extracted from the state space, as defined in the following.

#### 2.3.2. State Spaces

The *Quattracker IMU* uses a constant angular velocity and a constant acceleration model [43]. Thus, the angular velocity and the linear acceleration are required in the state. Assuming a rigid and known I2S calibration, the IMU positions and orientations are sufficient to reconstruct the corresponding segment pose. Hence, for *n* segments the state at time *t* comprises:

(4) $$x_{t}={\left({\left\{I_{i,t}^{G},{\dot{I}}_{i,t}^{G}{\ddot{I}}_{i,t}^{G},q_{i,t}^{GI},\omega_{I,i,t}^{GI}\right\}}_{i=0}^{n-1}\right)}^{T}$$

The *Quattracker segment*, similarly to the *Chaintracker*, represents the kinematic variables in the segment frame. This leads to a dynamic model assuming constant linear and angular acceleration, while the angular acceleration is required as part of the state:

(5) $$x_{t}={\left({\left\{S_{i,t}^{G},{\dot{S}}_{i,t}^{G}{\ddot{S}}_{i,t}^{G},q_{i,t}^{GS},\omega_{S,i,t}^{GS},{\dot{\omega }}_{S,i,t}^{GS}\right\}}_{i=0}^{n-1}\right)}^{T}$$

Note, the introduction of $\dot{\omega }$ is a consequence of the need for ${\ddot{I}}^{G}$ in Equation (1), since:

(6a) $$\begin{array}{ccc} I_{i,t}^{G} & = & S_{i,t}^{G}+R_{i,t}^{GS}I_{i,t}^{S} \end{array}$$

(6b) $$\begin{array}{ccc} \Rightarrow {\dot{I}}_{i,t}^{G} & = & {\dot{S}}_{i,t}^{G}+{\dot{R}}_{i,t}^{GS}I_{i,t}^{S}+R_{i,t}^{GS}\underset{=0}{\underset{︸}{{\dot{I}}_{i,t}^{S}}} \end{array}$$

(6c) $$\begin{array}{ccc} {\dot{I}}_{i,t}^{G} & = & {\dot{S}}_{i,t}^{G}+R_{i,t}^{GS}S\left(\omega_{S,i,t}^{GS}\right)I_{i,t}^{S} \end{array}$$

(6d) $$\begin{array}{ccc} \Rightarrow {\ddot{I}}_{i,t}^{G} & = & {\ddot{S}}_{i,t}^{G}+{\dot{R}}_{i,t}^{GS}S\left(\omega_{S,i,t}^{GS}\right)I_{i,t}^{S}+R_{i,t}^{GS}S\left({\dot{\omega }}_{S,i,t}^{GS}\right)I_{i,t}^{S} \end{array}$$

#### 2.3.3. Dynamic Models

The dynamic model for the *Quattracker IMU* is:

(7) $$x_{t+T}=\;\;\;\left[\begin{array}{c} I_{i,t+T}^{G}\\ {\dot{I}}_{i,t+T}^{G}\\ {\ddot{I}}_{i,t+T}^{G}\\ q_{i,t+T}^{GI}\\ \omega_{I,i,t+T}^{GI}\\ \vdots \end{array}\right]\;\;\;=\;\;\;\left[\begin{array}{c} I_{i,t}^{G}+T{\dot{I}}_{i,t}^{G}+\frac{T^{2}}{2}{\ddot{I}}_{i,t}^{G}\\ {\dot{I}}_{i,t}^{G}+T{\ddot{I}}_{i,t}^{G}\\ {\ddot{I}}_{i,t}^{GI}+Te_{i,t}^{\hat{p}}\\ q_{i,t}^{GI}⊙exp\left(\frac{T}{2}\omega_{I,i,t}^{GI}\right)\\ \omega_{I,i,t}^{GI}+Te_{i,t}^{\hat{\omega }}\\ \vdots \end{array}\right]$$

where ⊙ is the quaternion product, exp denotes the quaternion exponential and *T* is the sampling time. Note, the vertical dots indicate that these variables are given for each segment i∈{0,…,n-1}.

Equation (7) is built similarly for the *Quattracker segment*, by applying the following two modifications: (1) replace $I^{G}$ with $S^{G}$ and (2) consider $\dot{\omega }$ in the rotational update by utilizing Equation (B4) (Appendix B). Thus, Equation (7) can be extended as follows:

(8) $$x_{t+T}=\;\;\;\left[\begin{array}{c} S_{i,t+T}^{G}\\ {\dot{S}}_{i,t+T}^{G}\\ {\ddot{S}}_{i,t+T}^{G}\\ q_{i,t+T}^{GS}\\ \omega_{S,i,t+T}^{GS}\\ {\dot{\omega }}_{S,i,t+T}^{GS}\\ \vdots \end{array}\right]\;\;\;=\;\;\;\left[\begin{array}{c} S_{i,t}^{G}+T{\dot{S}}_{i,t}^{G}+\frac{T^{2}}{2}{\ddot{S}}_{i,t}^{G}\\ {\dot{S}}_{i,t}^{G}+T{\ddot{S}}_{i,t}^{G}\\ {\ddot{S}}_{i,t}^{GI}+Te_{i,t}^{\hat{p}}\\ q_{i,t}^{GS}⊙\exp\left(\frac{T}{2}\omega_{S,i,t}^{GS}+\frac{T^{2}}{4}{\dot{\omega }}_{S,i,t}^{GS}\right)\\ \omega_{S,i,t}^{GS}+T{\dot{\omega }}_{S,i,t}^{GS}\\ {\dot{\omega }}_{S,i,t}^{GS}+e_{i,t}^{\dot{\hat{\omega }}}\\ \vdots \end{array}\right]$$

The following equations obtain the quantities required for the measurement models (as defined in Section 2.3.1) from the above state using the I2S calibrations:

(9a) $$\begin{array}{cc} {\ddot{I}}_{i,t}^{G} & ={\ddot{S}}_{i,t}^{G}+R_{i,t}^{GS}\left[S\left(\omega_{S,i,t}^{GS}\right)S\left(\omega_{S,i,t}^{GS}\right)+S\left({\dot{\omega }}_{S,i,t}^{GS}\right)\right]I_{i}^{S} \end{array}$$

(9b) $$\begin{array}{cc} q_{i,t}^{GI} & =q_{i,t}^{GS}⊙q_{i}^{SI} \end{array}$$

(9c) $$\begin{array}{cc} \omega_{I}^{GI} & =q_{i}^{IS}⊙\omega_{S,i,t}^{GS}⊙q_{i}^{SI}={\left(R_{i,t}^{SI}\right)}^{T}\omega_{S,i,t}^{GS}. \end{array}$$

#### 2.3.4. Constraints

Constraints within an EKF are commonly implemented as measurement models [44,45]. In this work, in order to reduce drifts, constraints are used to ensure the segments staying connected at the joints. The following models are reformulated biomechanical constraints from [19]. Let $S_{i,t}^{G},S_{j,t}^{G}$ be the global position of two connected segments $S_{i},S_{j}\in S_{J_{k}}$ at time *t*. Let $R_{i,t}^{GS}$ and $R_{j,t}^{GS}$ be their orientations. Given $p_{i}^{S}\in R^{3}$, an endpoint of $S_{i}$, and $p_{j}^{S}\in R^{3}$, the corresponding point in $S_{j}$ (cf. Figure 1b), the measurement model that ensures connection of the two segments is:

(10) $$0=S_{i,t}^{G}+R_{i,t}^{GS}p_{i}^{S}-\left(S_{j,t}^{G}+R_{j,t}^{GS}p_{j}^{S}\right)-e_{i,t}^{p}$$

By exchanging the point of segment *j* by a constant point in the global system $P^{G}$, the model:

(11) $$0=S_{i,t}^{G}+R_{i,t}^{GS}p_{i}^{S}-P^{G}-e_{i,t}^{G},$$

ensures that the point $p_{i}^{S}$ stays close to the global point $P^{G}$ for all time steps. This is used in the evaluation for fixing the position of the root segment’s origin.

#### 2.3.5. Initialization

Given the first set of measurements, the TRIAD method [46] is used to determine the initial IMU orientations $q_{i,0}^{GI}$. For the *Quattracker IMU* the obtained orientations can be introduced into the state directly, whereas the *Quattracker segment* requires these to be transformed into the segment orientations using the I2S calibrations. After initializing the orientations, in a second step, it is ensured, that Equation (10) is fulfilled for the correctly rotated segments. The initial angular and linear velocities and accelerations are set to zero. The state of the *Chaintracker* comprises the variable angles and angle derivatives of the given kinematic chain (Appendix A). The angle derivatives are initialized with zero, while the angles are calculated from the initial quaternions $q_{i,0}^{GI}$ via inverse kinematics. This is done by minimizing:

(12) $$\underset{x_{c}}{\mathrm{argmin}}\sum_{i=0}^{n-1}{∥\left(R_{i,0}^{GI}rot{\left(f_{i}\left(x_{c}\right)\right)}^{T}-I_{3\times 3}\right)∥}_{F}$$

where *n* is the number of IMUs, ${\left||\;\cdot \;|\right|}_{F}$ denotes the Frobenius norm and $rot{\left(f\left(x_{c}\right)\right)}^{T}$ extracts the global orientations from the chain state $x_{c}$ (Appendix A).

### 2.4. Sliding Window Optimization

While the presented EKF approaches only maintain a state for a single time step and process one IMU data set at a time, the optimization-based approach uses windows or batches of IMU data sets to compute a corresponding batch of states (see Equation (13)). The method is based on [19], however, extends this offline optimization approach to an online-capable method by introducing the window mechanism and appropriately adapting the cost function (cf. Section 2.4.2). As suggested in [19], both the IMU and segment poses are estimated in the state. This relaxes the rigidity assumption concerning the I2S calibrations. Moreover, in contrast to the EKF approaches, the dynamic model takes the IMU data as control input (see Appendix C). This results in the IMU velocities being estimated in the state, while the need for estimating the linear accelerations and angular velocities is avoided. The state is composed as follows:

(13) $$x^{b}={\left({\left\{{\left\{I_{i,t}^{G},{\dot{I}}_{i,t}^{G},q_{i,t}^{GI},S_{i,t}^{G},q_{i,t}^{GS}\right\}}_{i=0}^{n-1}\right\}}_{t=0}^{w-1}\right)}^{T}$$

where *w* is the window size, *n* the number of segments and *b* is the batch number. For every optimization step, an overlap ov with w>ov≥1 is defined. Setting ov=1 results in a delayed tracking, where the delay depends on the setting of *w*, while ov=w-1 leads to online tracking using moving horizon estimation. Here, for each new time step, the latest IMU data is added and the oldest is discarded.

The optimization-based approach comes down to solving a weighted least-squares problem for each batch of data, where the different terms in the cost function are obtained by rearranging the stochastic model equations so that the noises are isolated on the left-hand side:

(14) $$\begin{array}{c} \min_{x^{b}}\sum_{t=0}^{w-1}\sum_{i=0}^{n-1}\left(\underset{12S\;\text{calibrations},\;\text{Appendix C}}{\underset{︸}{{∥e_{i,t}^{cq}∥}_{{\left(\Sigma^{cq}\right)}^{-1}}^{2}+{∥e_{i,t}^{cp}∥}_{{\left(\Sigma^{cp}\right)}^{-1}}^{2}}}\right.\left.+\underset{\text{motion}\;\text{model},\;\text{Appendix C}}{\underset{︸}{{∥e_{i,t}^{\hat{p}}∥}_{{\left(\Sigma^{\hat{p}}\right)}^{-1}}^{2}+{∥e_{i,t}^{\dot{\hat{p}}}∥}_{{\left(\Sigma^{\dot{\hat{p}}}\right)}^{-1}}^{2}+{∥e_{i,t}^{\hat{q}}∥}_{{\left(\Sigma^{\hat{q}}\right)}^{-1}}^{2}}}\right.\\ \left.+\underset{\text{prior},\;\text{Equation}\;(11)}{\underset{︸}{∥e_{i,t}^{G}{∥}_{{\left(\Sigma^{G}\right)}^{-1}}^{2}}}+\underset{\text{magnetometer}\;\text{model},\;\text{Equation}\;(3)}{\underset{︸}{∥e_{i,t}^{m}{∥}_{{\left(\Sigma^{m}\right)}^{-1}}^{2}}}\right)\\ +\sum_{i=0}^{n-1}\left(\underset{\text{initialization},\;\text{Section}\;2.4.2}{\underset{︸}{∥e_{i,t}^{q0}{∥}_{{\left(\Sigma^{q0}\right)}^{-1}}^{2}}}\right)+\sum_{t=0}^{w-1}\sum_{J_{k}\in J}\left(\underset{\text{biomechanical}\;\text{constraints},\;\text{Section}\;2.4.1}{\underset{︸}{∥\left(e_{k,t}^{p}\right){∥}_{{\left(\Sigma^{p}\right)}^{-1}}^{2}}}\right) \end{array}$$

The following sections explain the individual terms in more detail. For the sake of completeness, the terms already proposed in [19] are provided in the Appendix C. Note, compared to [19], the prior and magnetometer model term were added in order to enable fair comparison with the EKF-based methods.

#### 2.4.1. Biomechanical Constraints

The biomechanical constraints (connected joints) are based on the re-arrangement of Equation (10). In contrast to [19], where these are integrated as hard constraints, these are here included as terms into the cost function. Thus, an unconstrained weighted least squares problem is obtained, which can be solved using standard nonlinear least-squares methods, for instance the Gauss Newton or Levenberg Marquardt method [47,48].

#### 2.4.2. Initialization

The initialization of the IMU orientations can be obtained by minimizing $\forall I_{i}\in I$

(15) $$e_{i,t}^{q0}=\left\{\begin{array}{cc} 2\log\left(q_{i,w-1}^{IG,b-1}⊙q_{i,0}^{GI,b}\right) & \text{for}\;b>0\\ 2\log\left(q_{i,init}^{IG}⊙q_{i,0}^{GI,b}\right) & \text{else}\; \end{array}\right.$$

This penalizes sudden changes of the estimated IMU orientations for the overlap of b-1 and *b*, which is a required adaptation for the proposed online-capable method compared to [19]. Note, in a moving horizon context this term corresponds to the arrival cost for the variables. Here, log denotes the quaternion logarithm. Note, for b=0, similarly to the EKF methods, the initial quaternions $q_{i,init}^{GI}$ are obtained using the TRIAD algorithm.

### 2.5. Summary and Overview

After the different methods have been described in detail, Table 1 provides a comparative summary of their characteristics.

It also adds information concerning their computational complexities, which mainly depend on the number of estimation variables per time step and the solution method. Also note that a higher redundancy in the formulation of the estimation problem results in an increase in the number of tuning parameters.

The *Chaintracker*, in contrast to all other methods, only models rotational quantities in the state. This is due to using the kinematic chain model where positions and translations are implicitly given. All EKF-based methods assume the I2S calibrations being error-free, while the *Optitracker* assumes Gaussian distributed zero-mean errors being present in the I2S calibrations. The *Quattracker IMU* and the *Quattracker segment* differ in the coordinate frame, in which the state variables are given. The former is IMU centered, while the latter, similarly to the *Chaintracker*, is segment centered. The segment centered formulation results in the need for keeping the segments’ angular accelerations in the state and, thus, taking these into account in the dynamic model. Hence, the *Quattracker segment* and the *Chaintracker* only differ in the biomechanical model representation (kinematic chain vs. free segments) and the orientation parametrization (quaternions vs. joint angles). The *Optitracker*, in contrast to the EKF-based methods, keeps both the segment poses and the IMU poses in the state. Moreover, it takes the IMU data as control input to the dynamic model. With its comparably high level of redundancy in both spatial and temporal dimension, as well as, its iterative estimation method, the *Optitracker* has a significantly higher computational complexity than the other methods.

### 2.6. Evaluation Setup

The overall goal of the evaluation was to answer the question, which of the proposed sensor fusion schemes (*Quattracker segment*, *Quattracker IMU*, *Optitracker*), in comparison to the existing method (Chaintracker, here considered as baseline), provides the best basis for developing a truly robust online inertial body tracking method. Based on the argumentation in Section 1, sensitivity to the selected model calibration errors and dependence on magnetometer usage was in the focus. The evaluation was performed as a case study with a kinematic model comprising three rigid segments with known lengths, which are connected through two three DoF joints. The root joint was assumed fixed in space, with three rotational DoFs. In terms of motion sequences, moderate and fast motion was considered, in order to compare the performances of the different sensor fusion methods *w.r.t.* the above mentioned criteria also in relation to the motion agility (cf. [20]). General complex (i.e., containing simultaneous variations in all joint DoFs) motion was chosen instead of planar motions or specific activities, since the focus was on the comparison of the different sensor fusion methods under general conditions, rather than on their ability to represent specific motion patterns. Further explanations are provided below. Within this case study, we investigated two scenarios: (1) a real data scenario and (2) a simulation scenario with systematically introduced calibration errors. Both were investigated under two test configurations: (1) using magnetometer information (w/mag) and (2) dropping the magnetometer information, i.e., dropping the magnetometer measurement model in Equation (18) for all sensor fusion methods (w/o mag). The two scenarios, together with the used data sequences, as well as, the measures of performance are described in the following.

#### 2.6.1. Real Data Scenario

The goal of the real data scenario was to assess and compare the overall performances of the different sensor fusion methods under realistic conditions. Here, different error sources are unavoidably present and cannot be reliably separated, as also discussed in [9]. The major ones are therefore characterized in Section 2.6.2. Hence, the real data setup served for gathering tendencies concerning the overall robustness of the different sensor fusion methods under realistic conditions, while the influence of the considered model calibration errors were assessed in simulation (cf. Section 2.6.3). The results of the real data scenario are summarized in Section 3.1.

For data collection, the following systems were used: the NaturalPoint OptiTrack system with 12 Prime 13 cameras, operated with the Motive software (Version 1.8) (NaturalPoint, Inc., Corvallis, OR, USA) [50] and the XSens Link system, operated with the MVN Studio BIOMECH software (Version 4.1) (Xsens, Enschede, The Netherlands) [51]. The former served as reference, while the latter provided the real IMU data. Both systems recorded data at 120 Hz.

**Figure 2:**
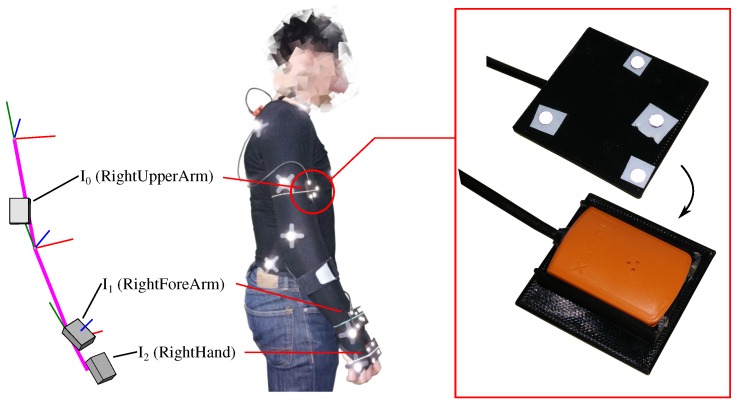
Capturing setup for the real data scenario. In the picture on the left, the segment coordinate systems are associated to the proximal ends of the segments. Note, the axes are orthogonal and only roughly aligned with the anatomical axes of rotation through the skeleton fitting of the optical system as described in Section 2.6.1. Precise alignment with the anatomical axes was not in the focus of this study. In the N-pose, for the right arm, the *x*-axes are chosen perpendicular to the frontal plane pointing anterior, the *y*-axes are perpendicular to the transverse plane pointing along the segments in the direction from the distal to the proximal ends and the *z*-axes are perpendicular to the sagittal plane pointing lateral. The picture also indicates the initial arm configuration for *real-slow* and *real-fast*.

A healthy male (age: 30 years, height: 1.76 m) was equipped with three IMUs at the right upper arm, forearm and hand (cf. Figure 2). Each IMU was mounted into a special casing with optical markers (4 mm diameter) attached to it, in order to ensure a rigid connection between IMU and marker rigid body. Additional markers (12 mm diameter) were placed at anatomical landmarks in order to enable skeleton fitting using the Motive software (upper body model, 25 markers). The cameras were arranged in a small volume in order to enable continuous tracking of the small markers.

**Figure 3:**
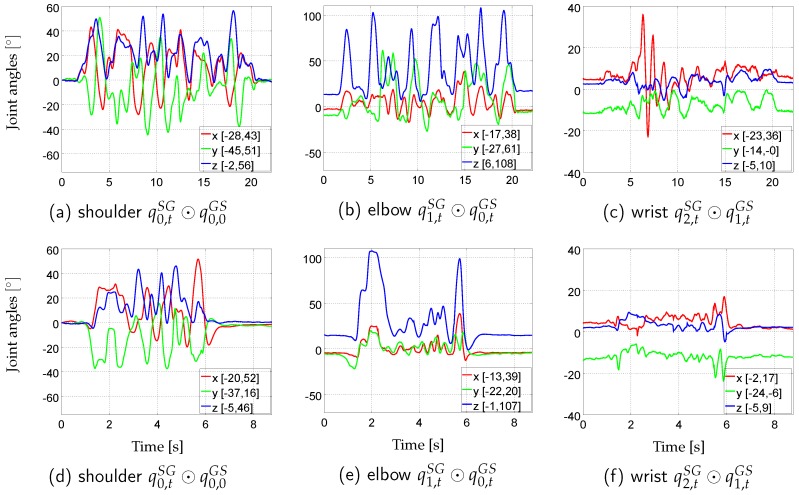
Real data scenario: Euler angle sequences ($z,x^{\prime },y^{\prime \prime }$ convention) and ranges of motion, [minimumangle,maximumangle] (each provided in degree), of *real-slow* (**a**–**c**) and *real-fast* (**d**–**f**). The segment axes and initial segment orientations are as shown in Figure 2. Note, the shoulder angles (left column) are represented *w.r.t.* to the initial upper arm configuration $q_{0,0}^{GS}$, rather than *w.r.t.* the global frame, in order to cancel out the unknown heading offset for easier interpretation.

With this setup, two evaluation data sequences with complex moderate and fast human motion were recorded. For this, the subject was asked to perform a movement, which introduces simultaneous variations in all DoFs of the right arm, while keeping the shoulder stationary, once at moderate speed and once at fast speed with fast speed changes. The movements also had to be performed anterior to the frontal plane and with the hand roughly below the shoulder height in order to be well captured by the optical system. The resulting data sequences, subsequently denoted *real-slow* and *real-fast*, both contain eight-shaped movements at respective speeds, smooth parts reminding of reaching and steering in the case of *real-slow*, and agile parts with quick starts and stops, as well as, parts reminding of sportive movements, such as boxing, in the case of *real-fast*. In Figure 3, the two data sequences are illustrated in terms of Euler angles per time step and respective ranges of motion for each joint DoF. The ranges of motion are comparable between the sequences, besides *sim-fast* covering smaller ranges for rotations around the *y*-axes of shoulder and elbow (external rotations of upper and forearm). The ranges of motion for the hand DoFs are the smallest among the considered joints. Videos showing re-simulations of *real-slow* and *real-fast* (called *sim-slow* and *sim-fast*) are available as supplementary files.

For each time step *t*, the data sequences contain the orientations and positions of the marker rigid bodies (as generated with the Motive software) in the world reference frame *O* of the optical system, the joint positions and orientations of the fitted skeleton and the IMU data, ${\left\{y_{i,t}^{\omega },y_{i,t}^{a},y_{i,t}^{m}\right\}}_{i=0}^{2}$. The joint data was only used to obtain the I2S calibrations (see below). In addition to the Euler angles, the measured peak accelerometer and angular velocity 2-norms are shown in Table 2.

Similarly to [52], temporal synchronization of optical and inertial data was done by maximizing the correlation between the angular velocities measured by the IMUs and the angular velocities derived from the marker rigid body orientations. The latter were calculated according to [53].

For spatial synchronization of the orientations obtained from the marker rigid bodies, $q_{i,t}^{OB}$, and the segment orientations estimated by the different sensor fusion methods, $q_{i,t}^{GS}$, the marker rigid bodies were transformed into the IMU reference frames using:

(16) $$\begin{array}{c} {\bar{q}}_{i,t}^{GS}=q^{GO}⊙q_{i,t}^{OB}⊙q_{i}^{BI}⊙q_{i}^{IS} \end{array}$$

Here, $q^{GO}$ and $q_{i}^{BI}$ denote the hand-eye orientations, which are the relative orientations aligning the global optical frame *O* with the global inertial frame *G*, and the marker rigid body frames $B_{i}$ with the IMU frames $I_{i}$. These were estimated from a data sequence with smooth motion, where the three IMUs were mounted on a stick, using the approach of [54]. Since the alignment approach in [54] is based on rotation sequences, the fused IMU orientations provided by the Link IMUs, $q_{i,t}^{GI}$, rather than the measured IMU data, and the orientations of the marker rigid bodies, $q_{i,t}^{OB}$, were used for calculation. Moreover, $q_{i}^{IS}$ refers to the I2S orientations. The I2S calibrations were calculated from a static recording of the subject being in a T-pose. For this, the fitted skeleton (i.e., the fitted 3D joint positions and orientations), the marker rigid body poses and the hand-eye orientations were used. The segment lengths were also derived from the fitted skeleton with: $∥S_{0}∥=0.28$ m, $∥S_{1}∥=0.23$ m, $∥S_{2}∥=0.08$ m (including only the palm). The resulting biomechanical model is shown in Figure 2 (left).

#### 2.6.2. Real Data Scenario: Discussion of Major Error Sources

This section provides a characterization of the major error sources in both the optical reference data and the IMU data. Such errors are unavoidably present and have to be taken into account for the analysis provided in Section 3.1. Equation (16) shows the different components involved into obtaining the reference segment orientations, which are subsequently used for measuring the performances of the different methods (cf. Section 2.6.4). Here, the errors in the orientations of the marker rigid bodies, $q_{i,t}^{OB}$, as obtained from the optical system, cannot be estimated. Note, the point tracking is claimed to provide sub-millimeter precision. The angular errors contributed through the hand-eye orientations, $q^{GO}$, $q_{i}^{BI}$, can be quantified for each IMU *i* to some extend based on the residual errors as calculated from each time step *t* of the data sequence used for calibration:

(17) $$E_{i,t}^{he}=2\;\mathrm{acos}\left(q^{GO}⊙q_{i,t}^{OB}⊙q_{i}^{BI}⊙q_{i,t}^{IG}\right)$$

Here, as mentioned above, $q_{i,t}^{IG}$ refers to the orientations obtained from the IMUs. The results are given in Table 3.

Concerning the I2S calibrations, $q_{i}^{IS}$, there are two aspects to consider. First, the errors in the calculated quantities cannot be estimated, since these were derived based on data provided by the optical system (see above). Second, due to soft tissue artifacts, these calibrations are in reality not rigid. In our study, the influence of soft tissue artifacts was not in focus and was minimized by well suited sensor placement, tight fixation and by ensuring a rigid connection between the marker rigid bodies used for calculating the reference segment orientations and the IMUs. Since the proposed sensor fusion methods all assume a kinematic model with rigid I2S calibrations (the *Optitracker* being the only method, which models Gaussian distributed I2S calibration errors), soft tissue artifacts, which can partly be interpreted as time-dependent changes of the I2S calibrations, still influence the estimated segment orientations, whereas the resulting error cannot be assessed.

The Link IMUs are intrinsically calibrated by the manufacturer, however, small gyroscope biases remain. These were approximated as mean gyroscope values from a static IMU data sequence and were subtracted from the angular velocities of the *real-slow* and *real-fast* sequences. The mean 2-norms of the accelerometer measurements under static conditions were 9.74 m/s${}^{2}$, 9.76 m/s${}^{2}$, 9.77 m/s${}^{2}$ for the three IMUs. These deviations from the local gravity strength [55] of 9.81 m/s${}^{2}$ were, however, not corrected.

Though no ferromagnetic objects where present in the capturing volume during recording, the measured magnetic field vectors indicate an inhomogeneous magnetic field. Table 4 shows the statistics of the magnetic field vector 2-norms and the angular deviations, $∡(y_{i,t}^{m},y_{mean,t})$, in the global frame for *real-slow* and *real-fast*. For each time step *t*, the latter were calculated as the normalized magnetic field measurements transformed into the global frame *G* using the optical reference data. More specifically, for *n* IMUs:

(18a) $$\begin{array}{ccc} y_{mean,t} & = & \frac{1}{n}\sum_{i=0}^{n}R_{i,t}^{GI}\frac{y_{i,t}^{m}}{∥y_{i,t}^{m}∥} \end{array}$$

(18b) $$\begin{array}{ccc} ∡(y_{i,t}^{m},y_{mean,t}) & = & \mathrm{acos}\left(y_{mean,t}\;R_{i,t}^{GI}\frac{y_{i,t}^{m}}{∥y_{i,t}^{m}∥}\right) \end{array}$$

where $R_{i,t}^{GI}$ were extracted from the optical reference data (cf. Equation (16)). Note that the resulting errors include the hand-eye calibration errors (cf. Table 3).

The table indicates stronger magnetic disturbances for $I_{1}$ and $I_{2}$ than for $I_{0}$, which is due to the varying distances of those IMUs from the floor, the latter causing an inhomogeneous magnetic field (as also observed in [56,57]). Since this work investigates the dependence of the proposed sensor fusion methods on magnetometer usage, rather than active compensation of magnetic disturbances, for evaluation, we also tested the different methods on real inertial data in combination with simulated magnetic field vectors (as also done in [9]). The latter were calculated by rotating the *x*-axis of the global frame *G* into the frame of each IMU *i* using:

(19) $$\begin{array}{c} y_{art,i,t}^{m}=R_{i,t}^{IG}{\left(1,0,0\right)}^{T} \end{array}$$

Here, again, the rotations $R_{i,t}^{GI}$ were extracted from the optical reference data (cf. Equation (16)).

#### 2.6.3. Simulation Scenario with Systematically Introduced Model Calibration Errors

The goal of the simulation scenario was to assess the influence of model calibration errors, i.e., I2S calibration and segment length errors, on the different sensor fusion methods, in the absence of other error sources. For this, three simulated data sequences were considered: a simulated data sequence based on an artificially generated smooth, but fast motion and re-simulations of *real-slow* and *real-fast*, which both contain human motion, as further explained below. These data sequences are subsequently referred to as *sim-fast-artificial*, *sim-slow* and *sim-fast*, respectively. The results of the simulation scenario are summarized in Section 3.2 and Section 3.3. The *sim-fast-artificial* sequence was simulated from an arm-like three segment kinematic chain model, which was parametrized using DH coordinates (cf. Table 5). The segment lengths were $∥S_{0}∥=0.4,∥S_{1}∥=0.4,∥S_{2}∥=0.2$, the I2S positions were $I_{0}^{S}={\left(0,0,0.3\right)}^{T}$, $I_{1}^{S}={\left(0,0,0.3\right)}^{T}$, $I_{2}^{S}={\left(0,0,0.1\right)}^{T}$ (all in m) and the I2S orientations were assumed identity.

**Figure 4:**
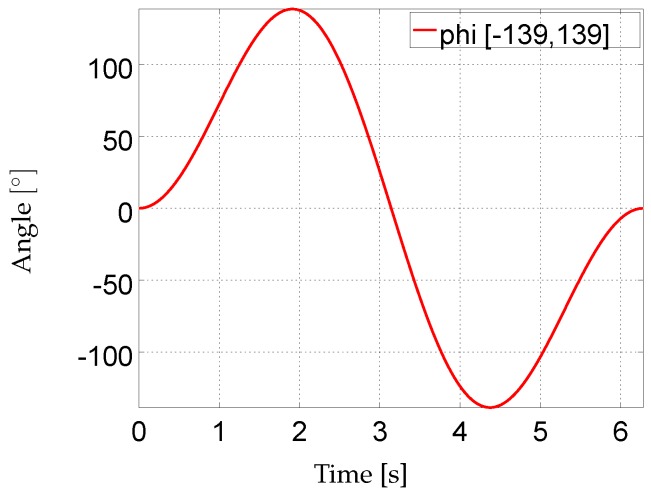
Simulation scenario: angle sequence applied to each rotational DoF of the three segment kinematic chain model (cf. Table 5) used for simulating *sim-fast-artificial*.

In order to animate this kinematic chain, $S_{0}$ was kept stationary at $W^{G}={\left(0,0,0.5\right)}^{T}$ and an angle sequence ${\left\{\phi \right\}}_{t=0}^{628}$ was generated using:

(20) $$\phi \left(\beta \left(t\right)\right)=\sin\left(\frac{\beta (t)}{2}\right)\sin\left(\beta \left(t\right)\right)\pi$$

with $\beta \left(t\right)=\frac{2\pi }{629}t$. This results in a sampling of one 2π period with a discretization of roughly 0.01 radians, where each step is assumed to correspond to a sampling time of 0.01s. This sequence was used for each rotational DoF $\alpha_{0},\ldots ,\theta_{2}$ and the resulting motion provides the ground truth segment orientations and positions for the *sim-fast-artificial* sequence. The angle sequence is visualized in Figure 4 and the motion resulting from applying this angle sequence to each rotational DoF is provided as a supplementary video. Based on the segment kinematics, the IMU trajectories are calculated by applying the assumed I2S calibrations. From this, IMU data was obtained using standard data differentiation [26] and, if applicable, by applying realistic sensor noises as detailed in Appendix D. The peak acceleration and angular velocity 2-norms resulting from the above described I2S calibrations for *sim-fast-artificial* are given in Table 6.

The *sim-fast-artificial* sequence provides simultaneous motion variations in all DoFs with large ranges of motion and with smoothly varying and periodically increasing and decreasing angular velocities, as well as direction changes. Comparing Figure 4 with Figure 3, the latter showing the Euler angle sequences and ranges of motion of *real-slow* and *real-fast*, *sim-fast-artificial*, sampling a range of motion of $\left[\pm 139^{\circ }\right]$, includes the maximum range of motion reached in the captured data sequences ($108^{\circ }$ elbow extension in *real-slow*), however, applies this to each rotational DoF. Moreover, comparing Table 6 with Table 2, *sim-fast-artificial* also reaches comparable peak angular velocity 2-norms to *sim-fast*. The peak accelerometer 2-norms are lower due to the artificial motion being more smooth, however, they are still considerably higher than in *real-slow*. Note, these accelerations depend on the assumed I2S positions. While *sim-fast-artificial* shares the mentioned characteristics with *real-fast*, as indicated above, it represents an artificial motion, which does neither resemble human motion patterns nor respect anatomical movement restrictions. However, it contains systematically sampled large ranges of motion and changing angular velocities in all DoFs, providing a challenging test case, as also visible in the supplementary video. Besides this, *sim-fast-artificial* also supports easy reproducibility by other researchers, without the need for sharing recorded data sequences, since the movement is based on an analytic function. Note, a similar sequence was also used in [40].

In contrast to the artificial motion in *sim-fast-artificial*, *sim-slow* and *sim-fast* represent re-simulations of the captured data sets *real-slow* and *real-fast*, i.e., they provide simulated IMU data for the human motions described in Section 2.6.1. As mentioned before, these movements are illustrated in the supplementary videos. For the simulation, the segment lengths, I2S calibrations and IMU trajectories were obtained from the optical system (cf. Section 2.6.1) and the IMU data was again obtained by differentiation. To ensure the IMU data to stay in feasible ranges, the IMU trajectories were low-pass filtered with a cut-off frequency of 10 Hz using a Butterworth filter of 4th order [58]. Table 2 shows the peak acceleration and angular velocity 2-norms of *sim-slow* and *sim-fast* in comparison to the respective real data sequences. Compared to *sim-fast-artificial*, the purpose of *sim-slow* and *sim-fast* is to provide IMU data based on human motion including respective limited joint DoFs and motion ranges, and, as already mentioned in Section 2.6.1, to provide slow and fast motion separately, in order to enable performance comparison of the different sensor fusion methods in relation to motion agility. Compared to *real-slow* and *real-fast*, the purpose of *sim-slow* and *sim-fast* is to provide IMU data for moderate and fast human motion with consistent ground truth and without errors, such as those described in Section 2.6.2. Moreover, the re-simulated data sequences enable comparison of the performances of the proposed sensor fusion methods between simulated and real data of the same motion.

Based on the above described motion sequences the influences of I2S calibration and segment length errors on the proposed sensor fusion methods were studied by systematically introducing respective model calibration errors when simulating the IMU data of the middle segment and comparing the estimated segment orientations with the ground truth data (cf. Section 2.6.4). The middle segment was chosen in order to enable assessing the error propagation behavior into neighboring segments. To this end, five calibration error types were introduced:

1. I2S position errors: $\Delta p_{I2S}^{along}\in \left[-0.2\;m,0.2\;m\right]$, i.e., position changes along the segment axis.
2. I2S position errors: $\Delta p_{I2S}^{out}\in \left[-0.2\;m,0.2\;m\right]$, i.e., position changes perpendicular to the segment axis.
3. Segment length variations: $∥\Delta S_{1}∥\in \left[-0.2\;m,0.2\;m\right]$.
4. I2S orientation errors: $\Delta q_{I2S}^{along}\in \left[-30^{\circ },30^{\circ }\right]$ along the bone, i.e., rotations around the segment axis associated to the IMU.
5. I2S orientation errors: $\Delta q_{I2S}^{out}\in \left[-30^{\circ },30^{\circ }\right]$ out of bone, i.e., rotations around the IMU axis perpendicular to the surface of the associated segment.

The ranges for the orientation errors are based on the findings in [22] concerning the trueness (deviation from reference) of established calibration methods. The reported maximum error of $26^{\circ }$ was slightly increased in order to account for the fact that larger errors are to be expected when calibration movements are performed by a subject autonomously. For obtaining the I2S positions and segment lengths, as described in Section 1.2, a variety of methods providing different and not well documented levels of reliability are in use. Hence, for this study, we determined the maximum I2S position error that could appear for the test subject by assuming the IMU to sit inside the joint, i.e., a 0.2 m shift along the forearm segment. We then used this value symmetrically for all translational error ranges, in order to be able to compare the influences of the different error types also relative to each other using a subsequently described normalized error measure (cf. Section 2.6.4). Note that these error ranges likely include the majority of errors that could potentially appear, e.g., due to varying body shapes and structures, heavy clothing or also failure of self-calibration methods, while they allow to compare the robustness of the different sensor fusion methods also under extreme conditions. Note, all considered model calibration errors were introduced separately in order to assess their influence in isolation.

The above error ranges were sampled with steps of $2^{\circ }$ and 0.02 m, respectively. In total, this summed up to 120 evaluations for each sensor fusion method.

#### 2.6.4. Error Measures

The performances of the different sensor fusion methods were evaluated based on the angular deviations of the estimated segment orientations, $q_{i,t}^{GS}$, from the reference segment orientations, ${\bar{q}}_{i,t}^{GI}$. For the real data scenario, the latter were calculated from the optical data according to Equation (16). For the simulation scenario, the segment orientations were available from the given motion sequence and can be considered as error-free ground truth. The angular deviations for each segment *i* at each time step *t* were calculated as [59]:

(21) $$E_{i,t}^{rel}=2\;\mathrm{acos}{\left({\bar{q}}_{i,t}^{GS}⊙q_{i,t}^{SG}\right)}_{w}$$

Here, ${\left(\;\cdot \;\right)}_{w}$ denotes the scalar part of the quaternion. The angular error statistics in terms of mean, standard deviation and maximum orientation errors over *K* segments and *N* time steps of a data sequence were calculated as:

(22a) $$\begin{array}{ccc} E^{mean} & = & \frac{1}{K}\sum_{i=0}^{K-1}\frac{1}{N}\sum_{t=0}^{N-1}E_{i,t}^{rel} \end{array}$$

(22b) $$\begin{array}{ccc} E^{sd} & = & \frac{1}{K}\sum_{i=0}^{K-1}\sqrt{\frac{1}{N-1}\sum_{t=0}^{N-1}{\left(E^{mean}-E_{i,t}^{rel}\right)}^{2}} \end{array}$$

(22c) $$\begin{array}{ccc} E^{max} & = & \max_{i=0,\ldots ,K-1}\max_{t=0,\ldots ,N-1}E_{i,t}^{rel} \end{array}$$

By setting K=1, the angular error statistics are calculated for each segment separately (K=3 considers all segments used in the evaluation). All angular errors are given in degrees.

Additionally to the above measures of performance, a *normalized range* error, $E_{\text{tracker}}^{norm}$, was also defined based on the mean angular errors, for quantifying the influence of model calibration errors on the different sensor fusion methods and simplifying the performance comparison. The normalized range error was defined as:

(23) $$\begin{array}{c} E_{\text{tracker}}^{norm}=\frac{\sum_{i=0}^{N_{var}-1}E_{\text{tracker}}^{mean}}{\max_{j=0,\ldots ,N_{test}-1}\sum_{i=0}^{N_{var}-1}E_{ref}^{mean}} \end{array}$$

Here, $N_{var}$ is the number of samples in the considered calibration error range, i.e., $N_{var}\;=\;30$ for I2S orientation errors and $N_{var}=20$ for I2S position and segment length errors. Moreover, $N_{test}$ denotes the number of tests considered ($N_{test}=6$ in the evaluation, i.e., three sequences, *sim-fast-artificial*, *sim-slow*, *sim-fast*, and two test conditions, w/mag, w/o mag). In Equation (23), the denominator scales the sum of mean errors, such that $E_{\text{tracker}}^{norm}$ is formulated relative to a reference $E_{\text{ref}}^{mean}$. For the latter, the *Chaintracker* was chosen, since this was considered as baseline for our work. Hence, for $E_{\text{tracker}}^{norm}=1$, the performance of the respective method is similar to the *Chaintracker*. Lower values indicate a better performance, higher errors indicate a worse performance.

## 3. Results

### 3.1. Tracking Performances on Real Data

This section presents the performance results of the different sensor fusion methods on real data, taking into account the inaccuracies as described in Section 2.6.1. The per segment angular error statistics (cf. Equation (22)) for the four sensor fusion methods (*Chaintracker*, *Quattracker segment*, *Quattracker IMU*, *Optitracker*) on *real-slow* and *real-fast* under both test conditions (w/mag, w/o mag) are summarized in Table 7. The following general tendencies could be observed: The *Optitracker* performed best *w.r.t.* mean errors, standard deviations and maximum errors on both data sequences and test conditions. The maximum error was $6.78^{\circ }$ and appeared at the forearm segment on *real-fast*. The mean errors were below $2.4^{\circ }$, and the standard deviations were below $1.4^{\circ }$ in all cases. Note the comparably low weight of the magnetometer measurements as used in the *Optitracker* (cf. Appendix D).

Compared to the *Optitracker*, the error margins of all EKF-based methods were considerably higher, if the real magnetometer measurements were used (Table 7, data rows 1–3 and 10–12, cf. Table 4). However, the error margins, in particular of the *Quattracker IMU*, were only slightly higher or comparable on *real-slow*, if the simulated magnetic field measurements (*sim. mag*) were used (data row 4–6). These error margins increased for all EKF-based methods on *real-fast*, although the mean errors remained below $4.3^{\circ }$. However, the maximum errors differed considerably. The *Quattracker segment* showed a maximum error of $14.94^{\circ }$ with the simulated magnetic fields and the *Chaintracker* showed a maximum error of $23.52^{\circ }$ with the real magnetometer measurements on *real-fast*. Moreover, *over all segments*, the *Chaintracker* had consistently (on both data sequences, w/mag and w/sim. mag) the highest mean errors and standard deviations, followed by the errors of the *Quattracker segment*, while the *Quattracker IMU* performed best among the EKF-based methods, indicating a higher robustness and accuracy for the IMU centered approaches in contrast to the segment centered ones. Also *over all segments*, all EKF-based methods showed a mean and maximum error increase on both data sequences, if the (undisturbed) magnetometer information was dropped. On *sim-slow*, mean errors, standard deviations and maximum errors increased on per segment basis. Note, in particular the maximum errors of the EKF-based methods increased on *real-slow* without using magnetometer information, but comparably less on *real-fast*. The *Optitracker*, in contrast, was not affected by dropping the magnetometer information, neither on *real-slow* nor on *real-fast*. In summary, the following indications could be observed: Under beneficial conditions (moderate motion, no magnetic disturbances), the EKF-based methods, in particular the *Quattracker IMU*, performed comparable or only slightly worse than the *Optitracker*. However, under fast motion changes (as present in *real-fast*) or magnetic disturbances, or when no magnetometer information was used, the performances of the EKF-based methods deteriorated, while the *Optitracker* was not considerably affected. Again, note the comparably low weight of the magnetometer measurements (cf. Appendix D). The higher robustness and accuracy of the *Optitracker*, however, comes at the cost of a considerable increase in computational complexity (cf. Table 1).

### 3.2. Tracking Performances on Simulated Data with Model Calibration Errors

**Figure 5:**
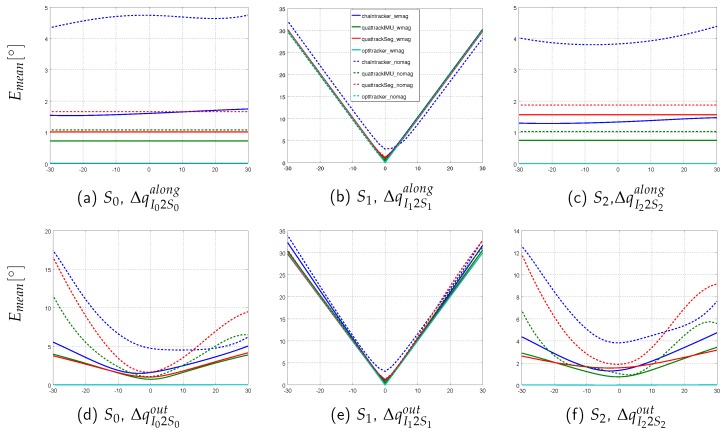
Simulation scenario: Per segment mean angular error distributions on *sim-fast* for along-bone and out-of-bone I2S orientation calibration errors (cf. Section 2.6.3).

**Figure 6:**
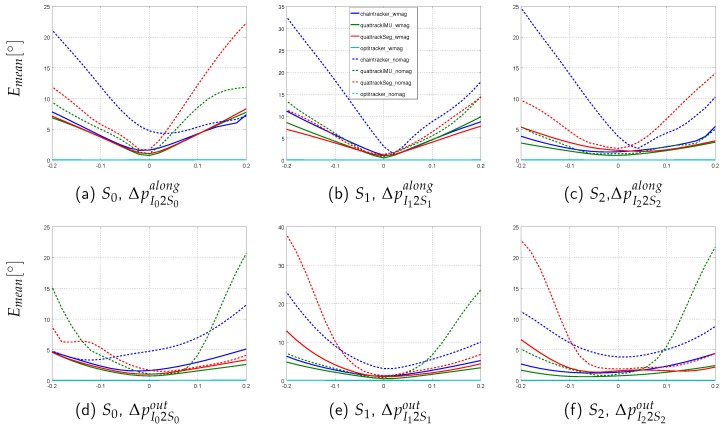
Simulation scenario: Per segment mean angular error distributions on *sim-fast* for along-bone and out-of-bone I2S position calibration errors (cf. Section 2.6.3).

**Figure 7:**
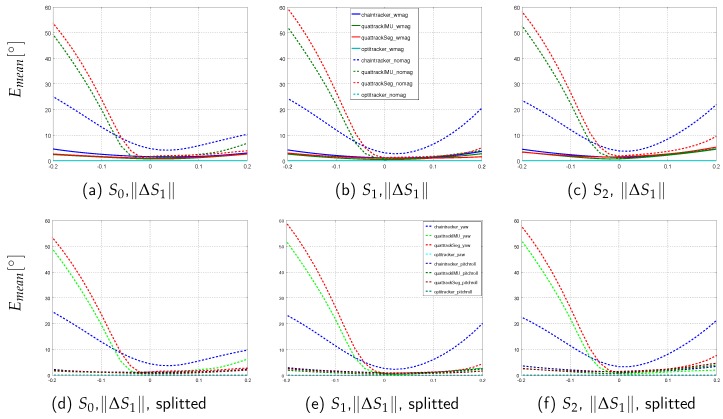
Simulation scenario: The upper row shows the per segment mean angular error distributions on *sim-fast* for segment length errors. The lower row shows the errors w/o magnetometers splitted into yaw and pitch/roll errors.

This section presents the performance results of the different sensor fusion methods on the simulated data sequences with systematically introduced model calibration errors. In order to evaluate the influence of these errors on the segment orientation estimation accuracy in isolation, perfect IMU data was here considered (cf. Section 3.3 for an evaluation with noisy data). The normalized range errors for the four sensor fusion methods on the three sequences (*sim-fast-artificial*, *sim-slow*, *sim-fast*) under both test conditions (w/mag, w/o mag) and for the five calibration error types ($\Delta p_{I2S}^{along}$,$\Delta p_{I2S}^{out}$,$∥\Delta S_{1}∥$,$\Delta q_{I2S}^{along}$,$\Delta q_{I2S}^{out}$) are summarized in Table 8. Moreover, the per segment mean angular errors for *sim-fast* are illustrated in Figure 5, Figure 6 and Figure 7. Here, *sim-fast* was chosen for a more detailed presentation, since it was the most challenging human motion considered. The results were analyzed *w.r.t.* the following aspects: (1) severity of angular error increases and error propagation from the affected middle segment $S_{1}$ to the previous ($S_{0}$) and subsequent ($S_{2}$) segments in relation to the error type; (2) aspect (1) w/o using magnetometers; (3) aspect (1) in relation to the motion agility, i.e., slow and fast motion and motion changes as available in *sim-slow*, *sim-fast* and *sim-fast-artificial*.

First, the results with magnetometers are described (Table 8a, solid plots in Figure 5, Figure 6 and Figure 7).

In Table 8a, the highest normalized range error of 0.51 was observed for the *Chaintracker* on *sim-fast-artificial* with error type $\Delta p_{I2S}^{out}$. Note that this was due to extremely high mean errors when applying an out-of-bone shift of above 0.18 m. This might exceed expected out-of-bone position errors, which, however, could be better handled by the other methods.

Besides this effect, the table shows, that I2S orientation errors had a higher influence on the estimation accuracy of the segment orientations than I2S position and segment length errors. This held for all data sequences and methods (lower errors in data rows 1–3 compared to 4–5). Moreover, all EKF-based methods showed an error increase from *sim-slow* to *sim-fast* and *sim-fast-artificial*, for all error types (data columns 5–8 compared to 9–12 and 1–4), while the *Optitracker* was not affected by the motion agility. In the majority of tests (combinations of data sequences and error types), the *Chaintracker* showed the highest errors, followed by the *Quattracker segment*, followed by the *Quattracker IMU*.

In addition to Table 8, Figure 5 illustrates the per segment distribution of mean angular errors for both orientation error types, $\Delta q_{I2S}^{along}$ and $\Delta q_{I2S}^{out}$, on *sim-fast*. As can be seen in Figure 5b,e, I2S orientation errors propagated at least linearly into the affected segment $S_{1}$. This held for both error types, $\Delta q_{I2S}^{along}$ and $\Delta q_{I2S}^{out}$, for all methods, and also for all data sequences (i.e., also for *sim-slow* and *sim-fast-artificial*, which are not shown in detail here). Note, a direct (linear) propagation is here expected, since all joints are modeled with three DoFs, without any explicit correction strategies, such as joint constraints. However, the different methods varied in the amount of error that was propagated into the neighboring segments (Figure 5a,c,d,f). While the *Optitracker* showed no noticeable error propagation (maximum mean error in $S_{0}$, $S_{2}$ on *sim-fast*: $0.03^{\circ }$), the EKF-based methods showed different behaviors. Regarding $\Delta q_{I2S}^{along}$, $S_{0}$ and $S_{2}$ showed errors that were rather constant over the whole range at different levels for all EKF-based methods. Regarding $\Delta q_{I2S}^{out}$, a real error propagation could be observed for all EKF-based methods, i.e., the mean errors in $S_{0}$ and $S_{1}$ varied depending on the introduced calibration errors. Comparable propagation behaviors were also observed for *sim-slow* and *sim-fast-artificial*, but with different error levels.

As already indicated above, for all EKF-based methods, the influence of I2S position errors on the segment estimation accuracy increased with higher motion agility (Table 8, data rows 1–3). In addition to the normalized range errors in the table, Figure 6 illustrates the per segment distribution of mean angular errors for both error types, $\Delta p_{I2S}^{along}$ and $\Delta p_{I2S}^{out}$, on *sim-fast*. Here, for all EKF-based methods, a clear influence on the affected segment (Figure 6b,e), as well as, a clear propagation into the neighboring segments (Figure 6a,c,d,f) could be observed. Note, while this observation held for both fast sequences, *sim-fast* and also *sim-fast-artificial*, the type of I2S position error, which caused the most severe normalized range error, was sequence dependent. While on *sim-fast*, $\Delta p_{I2S}^{along}$ caused the highest errors for all EKF-based methods, on *sim-fast-artificial*, $\Delta p_{I2S}^{out}$ caused the highest errors (Table 8, data rows 1–3, data columns 1–3 and 9–11). In contrast to the EKF-based methods, the *Optitracker* showed no noticeable error increases in any of the segments (maximum mean error on *sim-fast*: $0.04^{\circ }$), neither on *sim-fast*, nor on *sim-slow* or *sim-fast-artificial*. It could therefore be considered invariant to I2S position errors in the tested settings.

The same tendencies as for the I2S position errors could also be observed for all EKF-based methods for segment length errors, i.e., a normalized range error increase from *sim-slow* to *sim-fast* and *sim-fast-artificial* (Table 8, data row 3), an error propagation on the fast sequences (shown in Figure 7a–c for *sim-fast*), and also a sequence dependent level of influence of this error type. On *sim-fast*, $||\Delta S_{1}\left|\right|$ had a lower or comparable influence than/to both I2S position error types on the normalized range errors of the EKF-based methods (Table 8, data rows 1–3, data columns 9–11), and also on the per segment mean angular errors (maximum mean angular error over all segments and methods on *sim-fast*: $5.30^{\circ }$ by the *Quattracker segment*). In contrast, on *sim-fast-artificial*, $||\Delta S_{1}\left|\right|$ had a higher or comparable influence than/to $\Delta p_{I2S}^{along}$ (Table 8, data rows 1–3, data columns 1–3). Again, the *Optitracker* showed no noticeable error increases in any of the segments (maximum mean angular error on *sim-fast*: $0.04^{\circ }$), neither on *sim-fast*, nor on *sim-slow* or *sim-fast-artificial*.

In summary, in this study, I2S orientation errors (in particular $\Delta q_{I2S}^{out}$) had the most severe influence on the accuracy of the estimated segment orientations, for all methods and data sequences. For the EKF-based methods, the influence of translational error types (I2S position and segment length errors) was lower, but increasing with motion agility (i.e., higher for *sim-fast* and *sim-fast-artificial* compared to *sim-slow*). The specific influences of the different translational error types compared to each other were consistent between EKF-based methods, however, sequence dependent (different results for *sim-fast-artificial* compared to *sim-fast*), i.e., depending on the individual motion. The *Optitracker* was not considerably affected by those translational model calibration errors, independently of the data sequence. Over all data sequences, the *Quattracker IMU* performed best among the EKF-based methods and the *Optitracker* performed best among all methods.

In the following, the results without using magnetometer information are described (Table 8, dashed plots in Figure 5, Figure 6 and Figure 7). As for the real scenario, the results for the *Optitracker* were nearly identical under both test conditions and for all data sequences. Therefore, only the results for the EKF-based methods are further discussed in more detail.

Table 8 shows an increase of the normalized range errors for all EKF-based methods, when the (undisturbed) magnetometer information was dropped. This held for all data sequences and error types and is an expected behavior. An interesting fact is, however, that the error increase due to introduced I2S position and segment length errors was equally or more severe than the error increase due to introduced I2S orientation errors, in particular for the *Quattracker segment* and *Quattracker IMU* and for all data sequences (data columns 2–3, 6–7, 10–11). Moreover, Table 8, similarly to the test condition with magnetometers, showed an error increase from *sim-slow* to *sim-fast* and *sim-fast-artificial* for *Quattracker segment* and *Quattracker IMU* (data columns 6–7 compared to 2–3 and 10–11), while, again, the type of translational error that caused the highest normalized range error increase was sequence dependent. On *sim-fast*, segment length errors resulted in even higher normalized range errors than those caused by I2S orientation errors (data columns 10–11), while on *sim-fast-artificial*, $\Delta p_{I2S}^{out}$ had more influence among the translational error types. The *Chaintracker*, in contrast to *Quattracker segment* and *Quattracker IMU*, seemed to benefit from the agile motions in *sim-fast* and *sim-fast-artificial*, for both I2S orientation error types, but in a complementary manner for the translational error types (data column 5 compared to 1 and 9).

In addition to the table, Figure 6 and Figure 7 show exemplary for *sim-fast* that dropping the magnetometer information not only resulted in increased mean angular errors for the affected segment, but also in more severe error propagation into neighboring segments. This held for both I2S position and segment length errors. Concerning the I2S orientation errors (Figure 5), dropping the magnetometer information resulted in comparable performance for the affected segment (Figure 5b,e), while a higher propagation could be observed to the neighboring segments (Figure 5a,c,d,f).

A further peculiarity visible in the figures is the asymmetry of most of the error distributions over the error ranges when dropping the magnetometer information. Similar effects were observed for *sim-fast-artificial* and *sim-slow*. As exemplary shown in Figure 7d–f, the errors and, in particular the asymmetries, appeared mainly in the yaw direction. This is expected, since the yaw direction is the variable, where the magnetometer measurements provide reference information. Without this correcting information, the error in the yaw direction is supposed to be highly dependent on the individual configuration and motion leading to unpredictable results.

In summary, even on short motion sequences and noise-free IMU data, dropping the magnetometer information resulted in all EKF-based methods being more severely affected by model calibration errors. Moreover, translational error types (I2S position and segment length errors) gained more influence compared to I2S orientation errors, in particular for *Quattracker segment* and *Quattracker IMU* and increasing with motion agility. As for the test condition with magnetometers, the type of translational error that caused the most severe normalized range error was sequence dependent, but consistent between EKF-based methods. The *Optitracker*, in contrast to the EKF-based methods, was not affected by dropping the magnetometer information.

### 3.3. Tracking Performances on Simulated Data without Calibration Errors

When focusing on the results with no (or small) calibration errors in Figure 5, Figure 6 and Figure 7 (i.e., *x* around 0 in all graphs), the previously (real data scenario, simulation scenario w/mag) observed performance ranking (*Chaintracker*, *Quattracker segment*, *Quattracker IMU*, *Optitracker*) could be confirmed also for the simulation scenario w/o magnetometers, as exemplary shown in numbers for *sim-fast* in Table 9. The table provides the angular error statistics over all segments for the four sensor fusion methods on *sim-fast* under both test conditions (w/mag, w/o mag) and on noise-free and noisy IMU data. Hence, the table complements the results in the previous section for *sim-fast* by detailing the case, where no calibration errors were simulated and by providing results with noisy data. Note, comparable results as described below were obtained from *sim-slow* and *sim-fast-artificial*, but with overall lower error levels.

With noise-free IMU data (Table 9, data rows 1–2), the errors mainly depend on linearization, the degree to which the motion model fits the actual motion and the noise settings (cf. Appendix D). Concerning the former, the *Chaintracker* is expected to have the highest linearization errors, due to the angle parametrization, and the *Optitracker* is expected to have the lowest linearization errors, due to the nonlinear optimization. This is confirmed in the table. Moreover, the above performance ranking held for all test configurations and for mean errors, standard deviations and maximum errors. From the EKF-based methods, the *Quattracker IMU* provided the best results, with a mean error below $1^{\circ }$ for all test configurations. The *Optitracker* performed overall best, with a mean error below $0.4^{\circ }$ and a maximum error of $0.49^{\circ }$ over all test configurations.

For the EKF-based methods, noise did not have a significant influence (data rows 1–2 compared to 3–4). For the *Optitracker*, adding noise resulted in the error levels increasing according to the noise levels, with the above maximum error.

As previously observed, dropping the magnetometer information had no influence on the *Optitracker*. Also the *Quattracker segment* and *Quattracker IMU* showed an only slight error increase (mean, std, max) on this rather short *sim-fast* sequence, both on noise-free and noisy IMU data. Only the *Chaintracker* showed a more considerable error increase (mainly mean and std) when dropping the magnetometer information (data column 1). Note, that this effect might be reduced through specific tuning or by adding joint constraints (e.g., [20]). Since mainly the biomechanical model representation (kinematic chain versus free segments) and the rotation parametrization (joint angles vs. quaternions) differ between the two methods, this finding indicates that the combination of free segment model and quaternions is potentially more robust in the case of body motion tracking with unconstrained joints and without using magnetometer information.

## 4. Discussion and Conclusions

The goal of this work was to develop/identify sensor fusion methods, which robustly handle model calibration errors (I2S, segment length) and show potential for magnetometer-free operation. Concerning the latter, the conducted tests served mainly for excluding methods that do not cope well with missing reference information in the yaw direction, already on the rather short data sequences considered. Here, the motivation for this investigation was that potentially large model calibration errors have to be expected given today’s most widely used calibration techniques based on static poses, functional movements, manual measurements and assumptions. At the same time, magnetic disturbances have to be expected in man-made environments.

To this end, two newly developed/adapted sensor fusion methods (*Quattracker segment*, *Quattracker IMU*, *Optitracker*) were compared to an existing method (*Chaintracker*), regarding segment orientation tracking accuracy on complex moderate and fast captured and re-simulated human motion, as well as on fast artificial motion, in the presence and in the absence of model calibration errors, with and without using magnetometer information.

The findings concerning the influence of model calibration errors on the different methods, which held independently of the considered data sequences, can be summarized as follows: With (undisturbed) magnetometer information, I2S orientation errors clearly had a higher influence on segment orientation estimation accuracy than I2S position and segment length errors, which is an expected behavior. Both tested orientation error types, $\Delta q_{I2S}^{along}$ and $\Delta q_{I2S}^{out}$, resulted in at least linear error propagation into the affected segment. This held for all methods and was also expected, since no explicit compensation strategies, such as joint constraints were used (cf. [20]). For the EKF-based methods, different amounts of errors were also propagated into the orientation estimates of the neighboring segments, in particular for $\Delta q_{I2S}^{out}$, similarly for I2S position ($\Delta p_{I2S}^{along},\Delta p_{I2S}^{out}$) and segment length errors ($∥\Delta S_{1}∥$), increasing with motion agility. When dropping the magnetometer information, the influence of translational calibration error types (I2S position and segment length errors), relative to the influence of I2S orientation errors, increased considerably. From this, it can be concluded that I2S orientation errors represent the dominant source of the considered model calibration errors in magnetic-inertial human motion tracking (assuming undisturbed magnetometer information), while the importance of accurate I2S position and segment length calibration increases with motion agility and in the absence of reference information for the yaw direction (w/o mag). Note that in these cases all translational error types should be kept small, since their influences turned out to be motion dependent.

Further considering the results on real and simulated data without introduced calibration errors (cf. Section 3.1 and Section 3.3), it can also be stated for the newly developed EKF-based methods that dropping the magnetometer information (at least for the short time duration considered in the tests), resulted in acceptable accuracy loss, as long as no severe model calibration errors were present at the same time. The *Chaintracker*, however, showed the highest dependency on undisturbed magnetometer information and was overall more fragile.

The *Optitracker*, in contrast to all EKF-based methods, showed no considerable I2S orientation error propagation into neighboring segments, independently of the type of orientation error introduced ($\Delta q_{I2S}^{along}$, $\Delta q_{I2S}^{out}$). Moreover, it was nearly invariant *w.r.t.* I2S position and segment length errors, independently of motion agility and magnetometer usage. The latter was also confirmed in the real data scenario and in the simulation scenario without calibration errors. Note, the *Optitracker* also turned out to require no intensive tuning (cf. Appendix D).

Among the EKF-based methods, in the majority of test cases, the *Chaintracker* showed the highest (mean) errors (over all segments), followed by the *Quattracker segment*, followed by the *Quattracker IMU*. The *Optitracker* outperformed all EKF-based methods in all test cases, also showing lower standard deviations and maximum errors.

Hence, the segment centered biomechanical models (used by *Chaintracker* and *Quattracker segment*), which assume constant angular acceleration in the motion model (cf. [16] and Equation (8)), were overall outperformed by the IMU centered free segments biomechanical models (used by both, *Quattracker IMU* and *Optitracker*), while in the former case, the free segments model overall performed slightly better than the kinematic chain model. Moreover, the additional redundancy in terms of spatial and temporal estimation variables, in combination with the nonlinear optimization used by the *Optitracker* provided a considerable gain in robustness, in particular in the presence of disturbing effects as described above. Obviously, this comes at the cost of a significantly higher computational complexity, where, however, a parallelized version, similar to the one proposed in [32], might give remedy. Concerning the analysis above, the following aspects have to be noted: (1) the performances of the EKF-based methods could likely be improved through sequence dependent tuning, which is, however, not preferred for a practical system; (2) there exist other motion models, e.g., the decaying angular acceleration model [20], which were not investigated here.

Another important finding was that under beneficial conditions (moderate motion, no severe calibration errors, undisturbed magnetometer information), the *Quattracker IMU* provided the most comparable results to the *Optitracker*, but with considerably lower computational costs. Hence, the *Optitracker* might be best invested, if disturbing effects are expected in the considered application scenario, while, under beneficial conditions, the *Quattracker IMU* can be a computationally more efficient solution, which provides comparable performance. Based on this work, long-term magnetometer-free operation and robust handling of soft tissue artifacts remain parts of our future work.

## Figures and Tables

**Table 1:** Characteristics of the different sensor fusion methods: *n* denotes the number of segments and *w* is the window size used by the *Optitracker*. All tuning parameters are given in Appendix D. Note, $\theta_{i}$ represent the joint angles estimated by the *Chaintracker* and $\Sigma^{\ddot{\theta }}$ refers to the process noise covariances used in the dynamic model [16].

	*Chaintracker* (cf. [ 16])	*Quattracker segment*	*Quattracker IMU*	*Optitracker*
Estimation method	EKF	EKF	EKF	WLS
State	${\left({\left\{\theta_{i},{\dot{\theta }}_{i},{\ddot{\theta }}_{i}\right\}}_{i=0}^{n-1}\right)}_{t}^{T}$	${\left({\left\{S_{i,t}^{G},{\dot{S}}_{i,t}^{G}{\ddot{S}}_{i,t}^{G},q_{i,t}^{GS},\omega_{S,i,t}^{GS},{\dot{\omega }}_{S,i,t}^{GS}\right\}}_{i=0}^{n-1}\right)}^{T}$	${\left({\left\{I_{i,t}^{G},{\dot{I}}_{i,t}^{G}{\ddot{I}}_{i,t}^{G},q_{i,t}^{GI},\omega_{I,i,t}^{GI}\right\}}_{i=0}^{n-1}\right)}^{T}$	${\left({\left\{{\left\{S_{i,t}^{G},q_{i,t}^{GS},I_{i,t}^{G},{\dot{I}}_{i,t}^{G},q_{i,t}^{GI}\right\}}_{i=0}^{n-1}\right\}}_{t=0}^{w-1}\right)}^{T}$
Dimensions (state *s*, meas. vector *k*)	$x\in R^{9n}$, s=9n, k=7	$x\in R^{19n}$, s=19n, k=7	$x\in R^{16n}$, s=16n, k=7	$x\in R^{17n\times w}$, s=17nw
Motion model	1D const angular acc	3D const angular & linear acc	3D const angular vel; 3D const linear accel	IMU control input
Tuning parameters	$\Sigma^{\ddot{\hat{\theta }}},\Sigma^{a},\Sigma^{\omega },\Sigma^{m}$	$\Sigma^{\dot{\hat{\omega }}},\Sigma^{\hat{p}},\Sigma^{a},\Sigma^{\omega },\Sigma^{m},\Sigma^{p},\Sigma^{G}$	$\Sigma^{\hat{\omega }},\Sigma^{\hat{p}},\Sigma^{a},\Sigma^{\omega },\Sigma^{m},\Sigma^{p},\Sigma^{G}$	$\Sigma^{cq}$, $\Sigma^{cp}$, $\Sigma^{\hat{p}}$, $\Sigma^{\dot{\hat{p}}}$, $\Sigma^{\hat{q}}$, $\Sigma^{G},\Sigma^{m},\Sigma^{q0},\Sigma^{p}$
Complexity	$O(k^{2.4}+s^{2})$ [49]	$O(k^{2.4}+s^{2})$	$O(k^{2.4}+s^{2})$	$O(s^{3})$ (Gauss Newton method)
Biomech. model	chain	free segments	free segments	free segments
State coordinate system	segment centered	segment centered	IMU centered	IMU and segment centered

**Table 2:** Measured/simulated instantaneous peak acceleration (Acc) and angular velocity (Gyr) 2-norms for *real-slow*, *real-fast*, *sim-slow* and *sim-fast*.

Mode	Sequence →	Slow		Fast	
	Sensor →	Acc(m/s${}^{2}$)	Gyr (${}^{\circ }/s$)	Acc (m/s${}^{2}$)	Gyr (${}^{\circ }/s$)
Real	$I_{0}$	13.44	178.68	26.88	457.05
	$I_{1}$	16.87	394.68	75.24	957.29
	$I_{2}$	18.74	468.55	95.45	1031.31
Re-simulated	$I_{0}$	18.05	187.07	25.70	429.75
	$I_{1}$	22.07	395.27	77.20	968.35
	$I_{2}$	22.86	440.23	100.55	1030.76

**Table 3:** Mean (std,max) angular residual errors (cf. Equation (17)) for the hand-eye calibrations of each inertial measurement unit (IMU) $I_{i}$ as calculated on the data sequence used for calibration.

IMU	Residual Error
$I_{0}$	$1.14^{\circ }\left(0.57^{\circ },\;3.89^{\circ }\right)$
$I_{1}$	$2.28^{\circ }\left(0.90^{\circ },\;6.10^{\circ }\right)$
$I_{2}$	$1.51^{\circ }\left(0.77^{\circ },\;5.07^{\circ }\right)$

**Table 4:** Mean (std) of magnetic field vector 2-norms (upper values) and global angular deviations (lower values) for each IMU as calculated from the real data sequences.

	$I_{0}$	$I_{1}$	$I_{2}$
*real-slow*	0.92(0.00)	0.90(0.01)	0.90(0.02)
	$1.79^{\circ }\left(1.18^{\circ }\right)$	$3.66^{\circ }\left(2.41^{\circ }\right)$	$4.36^{\circ }\left(2.89^{\circ }\right)$
*real-fast*	0.92(0.00)	0.91(0.01)	0.91(0.02)
	$2.63^{\circ }\left(2.38^{\circ }\right)$	$4.45^{\circ }\left(5.60^{\circ }\right)$	$4.59^{\circ }\left(6.04^{\circ }\right)$

**Table 5:** Denavit-Hartenberg (DH) coordinates for the three segment kinematic chain model used for simulating the *sim-fast-artificial* data sequence. The angles $\alpha_{\left[0:5\right]}\left(t\right)$ and $\theta_{\left[0:2\right]}\left(t\right)$ are the Degrees of Freedom (DoFs) that are controlled via Equation (20). The Inertial Measurement Unit (IMU)-to-Segment (I2S) positions are given by translations along the bone, in *z*-direction relative to the segment origins (i.e., DH(z,0,0,0)). The initial chain configuration (pointing up opposite gravity) is illustrated on the right.

Segment ($S_{i}$)	*d*	*a*	$\alpha_{j}\left(t\right)$	$\theta_{j}\left(t\right)$	IMU	Image
$S_{0}$	0	0	$\alpha_{0}\left(t\right)$	$-\frac{pi}{2}$	None	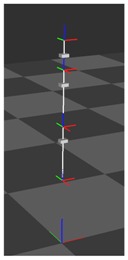
	0	0	$\alpha_{1}\left(t\right)$	$\frac{pi}{2}$	None	
	0	0	0	$\theta_{0}\left(t\right)$	None	
	0.4	0	0	0	z=0.3	
$S_{1}$	0	0	$\alpha_{2}\left(t\right)$	$-\frac{pi}{2}$	None	
	0	0	$\alpha_{3}\left(t\right)$	$\frac{pi}{2}$	None	
	0	0	0	$\theta_{1}\left(t\right)$	None	
	0.4	0	0	0	z=0.3	
$S_{2}$	0	0	$\alpha_{4}\left(t\right)$	$-\frac{pi}{2}$	None	
	0	0	$\alpha_{5}\left(t\right)$	$\frac{pi}{2}$	None	
	0	0	0	$\theta_{2}\left(t\right)$	None	
	0.2	0	0	0	z=0.1	

**Table 6:** Peak acceleration (Acc) and angular velocity (Gyr) 2-norms for *sim-fast-artificial*. For all sensors, the values vary smoothly between 0 and the peak values shown in the table.

Sequence →	*sim-fast-artificial*	
Sensor →	Acc (m/s${}^{2}$)	Gyr (${}^{\circ }/s$)
$I_{0}$	14.03	356.90
$I_{1}$	38.16	705.20
$I_{2}$	61.90	1047.99

**Table 7:**
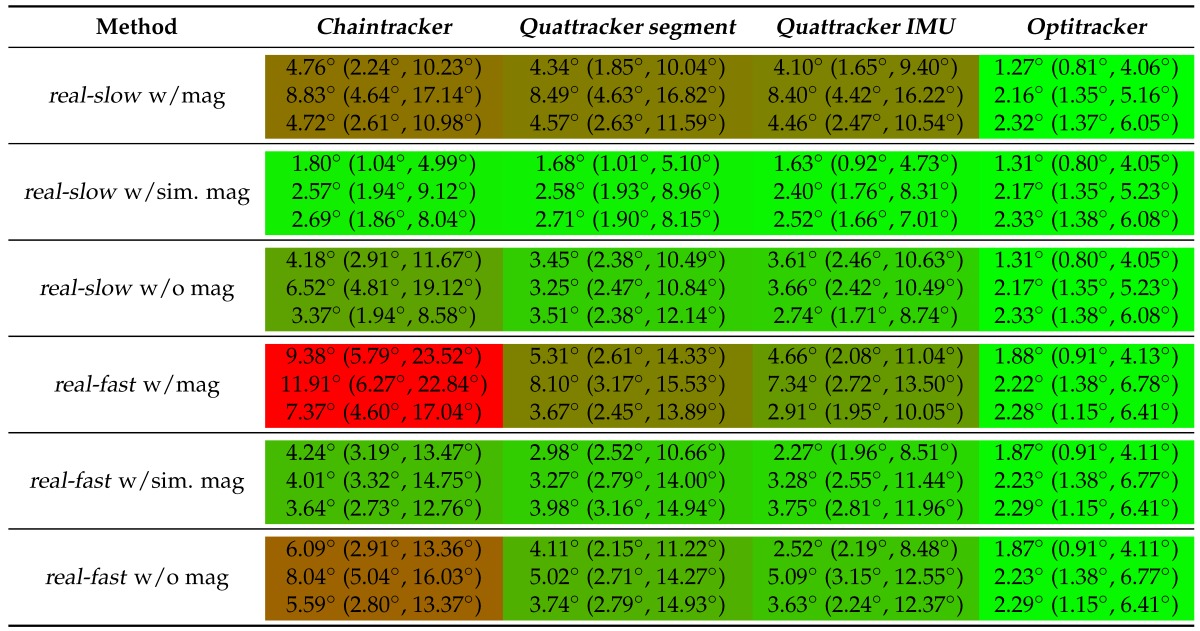
Real data scenario: mean (std; max) angular errors for each segment. Note, the color represents a linear interpolation of the mean error over all segments between red (maximum error) and green (minimum error). This helps comparing the performances of the different sensor fusion methods. Also note, w/mag refers to using the real magnetometer measurements, w/sim. mag refers to using the simulated magnetometer measurements (cf. Section 2.6.2) and w/o mag refers to dropping the magnetometer information.

**Table 8:**
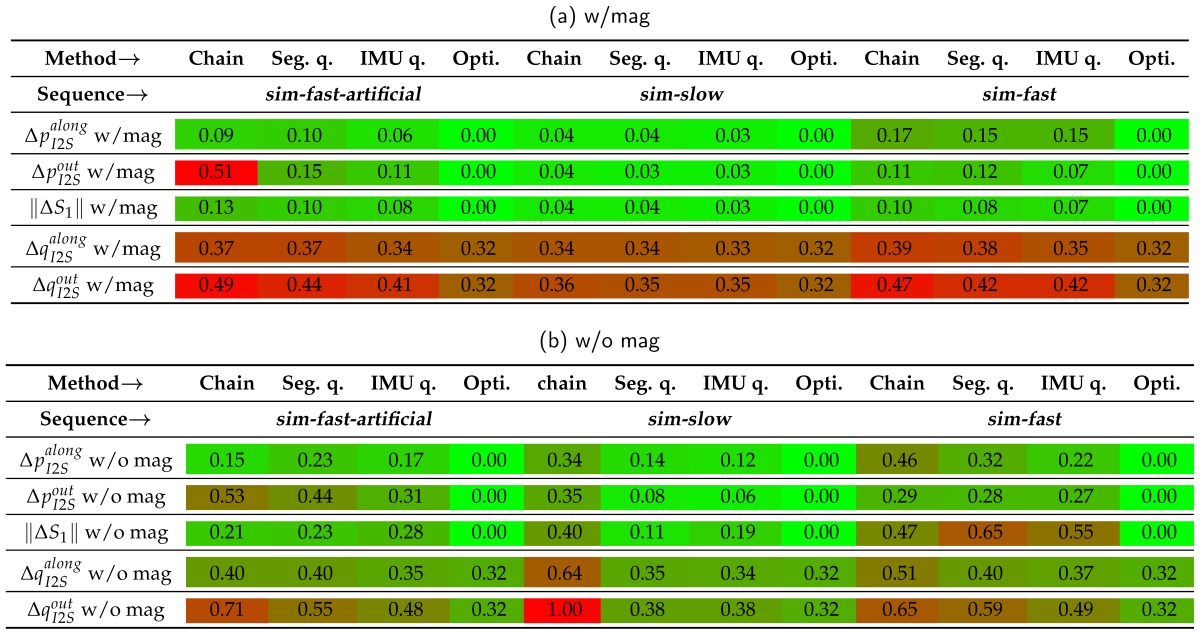
Simulation scenario: the normalized range error $E_{quot}^{tracker}$ (cf. Equation (23)) is shown for the different simulated model calibration errors and sensor fusion methods. The latter have the following shortcuts: *Chaintracker* (chain), *Quattracker segment* (Seg. q.), *Quattracker IMU* (IMU q.), *Optitracker* (opti.). Note, for each table separately, the color represents a linear interpolation of the error from red (maximum error) to green (minimum error).

**Table 9:** Simulation scenario without calibration errors (*sim-fast*): mean (std; max) angular errors over all segments on the two test configurations (w/mag, w/o mag), both on noise-free (perfect) and noisy IMU data.

Method	*Chaintracker*	*Quattracker segment*	*Quattracker IMU*	*Optitracker*
Noise-free w/mag	1.42${}^{\circ }$ (1.40${}^{\circ }$; 8.04${}^{\circ }$)	1.19${}^{\circ }$ (1.23${}^{\circ }$; 6.83${}^{\circ }$)	0.66${}^{\circ }$ (0.64${}^{\circ }$; 3.30${}^{\circ }$)	0.01${}^{\circ }$ (0.01${}^{\circ }$; 0.06${}^{\circ }$)
Noise-free w/o mag	3.50${}^{\circ }$ (2.57${}^{\circ }$; 9.45${}^{\circ }$)	1.57${}^{\circ }$ (1.45${}^{\circ }$; 7.52${}^{\circ }$)	0.97${}^{\circ }$ (0.65${}^{\circ }$; 3.36${}^{\circ }$)	0.01${}^{\circ }$ (0.01${}^{\circ }$; 0.06${}^{\circ }$)
Noise w/mag	1.46${}^{\circ }$ (1.39${}^{\circ }$; 8.09${}^{\circ }$)	1.22${}^{\circ }$ (1.21${}^{\circ }$; 6.83${}^{\circ }$)	0.69${}^{\circ }$ (0.62${}^{\circ }$; 3.28${}^{\circ }$)	0.40${}^{\circ }$ (0.05${}^{\circ }$; 0.49${}^{\circ }$)
Noise, w/o mag	3.73${}^{\circ }$ (2.68${}^{\circ }$; 9.76${}^{\circ }$)	1.55${}^{\circ }$ (1.40${}^{\circ }$; 7.42${}^{\circ }$)	0.95${}^{\circ }$ (0.65${}^{\circ }$; 3.31${}^{\circ }$)	0.40${}^{\circ }$ (0.05${}^{\circ }$; 0.49${}^{\circ }$)

## Supplementary Materials

The supplementary materials are available online at http://www.mdpi.com/1424-8220/16/7/1132/s1.

## Abbreviations

The following abbreviations are used in this manuscript:

DH: Denavit Hartenberg
DoF(s): Degree(s) of Freedom
EKF: Extended Kalman Filter
UKF: Unscented Kalman Filter
IMU: Inertial Measurement Unit
I2S: IMU-to-Segment
WLS: Weighted Least Squares

## Appendix A

A DH transformation is composed of a rotation around the *x*-axis (angle *α*), followed by two translations along the *x*- and the *z*-axis (a,d) and, finally, a rotation around the *z*-axis (angle *θ*). This results in the following homogeneous transformation:

(A1) $$DH\left(\theta ,d,a,\alpha \right)=\left(\begin{array}{cccc} \cos\theta & -\sin\theta \cos\alpha & \sin\theta \sin\alpha & a\cos\theta \\ \sin\theta & \cos\theta \cos\alpha & -\cos\theta \sin\alpha & a\sin\theta \\ 0 & \sin\alpha & \cos\alpha & d\\ 0 & 0 & 0 & 1 \end{array}\right)$$

These transformations are composed to a tree of paths:

(A2) $$DH_{path}\left({\left\{\theta_{i},d_{i},a_{i},\alpha_{i}\right\}}_{i=1}^{n}\right):=\prod_{i=1}^{n}H_{i}=\prod_{i=1}^{n}DH\left(a_{i},d_{i},\alpha_{i},\theta_{i}\right)$$

A kinematic chain is constructed by having an initial transformation $H^{GW}$, where *W* refers to the local world frame, i.e., the chain root. From there, paths lead to each IMU. Through this structure, each transformation is represented in the reference frame of its predecessor. Hence, the pose of the kth IMU in the global frame is given by:

(A3) $$H_{k}^{GI}=H^{GW}\prod_{i=1}^{k}\left(H_{i}\right)H_{k}^{SI}$$

According to the DH convention, per transformation, only one parameter may be variable. In our case, i.e., for human motion capturing with given segment lengths and I2S calibrations, only (joint) angles are estimation variables. A *chain state*, $x_{c}$, (i.e., a state space representing a kinematic chain) is therefore a set of variable angles. Consequently, the kth IMU pose in the global frame depends on the angles to its path:

(A4) $$H_{k}^{GI}=f_{k}\left(x_{c}\right)$$

where the right-hand side is given by Equation (A3). Note, $H^{GW}$ is here assumed given and fixed.

## Appendix B

The following notation is used: A quaternion is a 4-vector $q\in R,q={\left(q_{w},q_{x},q_{y},q_{z}\right)}^{T}$, where $q_{w}$ is the scalar part and $\left(q_{x},q_{y},q_{z}\right)$ is the vector part. Special quaternion sets are: $Q_{v}=\left\{q\in R^{4}:q_{w}=0\right\}$ and the set of unit quaternions (representing a rotation) $Q_{1}={\{q\in R:∥q∥}_{2}=1\}$. The identity quaternion is denoted as $I_{q}=\left(1,0,0,0\right)$.

The quaternion exponential is defined using a power series:

(B1) $$exp\left(q\right)=\sum_{n=0}^{\infty }\frac{q^{n}}{n!}$$

where the quaternion power is recursively defined as:

(B2) $$q^{n}=q⊙q^{n-1}=q^{n-1}⊙q,q^{0}=1$$

In the following equations, angular velocity *ω* and angular acceleration $\dot{\omega }$ are used in their quaternion formulation, i.e., (0,ω) and $\left(0,\dot{\omega }\right)$ respectively.

**Claim 1.** 
*The continuous-time differential equation describing the dynamic model of a unit quaternion rotating from frame B to A is:*

(B3) $${\dot{q}}_{t}^{AB}=\frac{1}{2}\omega_{A,t}^{AB}⊙q_{t}^{AB}$$

*Let $\omega_{A,t}^{AB},{\dot{\omega }}_{A,t}^{AB}$ be piece-wise constant between t and t+T, then:*

(B4) $$q_{t+T}^{AB}=\exp\left(\frac{T}{2}\omega_{A,t}^{AB}+\frac{T^{2}}{4}{\dot{\omega }}_{A,t}^{AB}\right)⊙q_{t}^{AB}$$

*is a consistent discrete-time dynamic model.*

**Proof of Claim 1.** Note, the frame subscripts and superscripts are omitted here for brevity. First, using the exponential formula for $exp(\frac{T}{2}\left(\omega +\frac{T}{2}\dot{\omega }\right))$ yields:

(B5) $$exp\left(\frac{T}{2}\left(\omega +\frac{T}{2}\dot{\omega }\right)\right)=I_{q}+\frac{T}{2}\omega +\frac{T^{2}}{4}\dot{\omega }+\frac{1}{2}{\left[\frac{T}{2}\omega +\frac{T^{2}}{4}\dot{\omega }\right]}^{2}+O\left(T^{3}\right)$$

To continue, the following multiplications need to be written out, since the binomial formulas do not hold for quaternions. Note that $\left(0,v\right)⊙\left(0,v\right)=\left(-v\;\cdot \;v,0_{3}\right)=\left(-v^{2},0_{3}\right)$:

(B6) $$\begin{array}{c} \frac{1}{2}{\left[\frac{T}{2}\omega +\frac{T^{2}}{4}\dot{\omega }\right]}^{2}=\frac{1}{2}\left(-\left[\frac{T}{2}\omega +\frac{T^{2}}{4}\dot{\omega }\right]\;\cdot \;\left[\frac{T}{2}\omega +\frac{T^{2}}{4}\dot{\omega }\right],0_{3}\right)\\ =\frac{1}{2}\left(-\frac{T^{2}}{4}\left[\omega +\frac{T}{2}\dot{\omega }\right]\;\cdot \;\left[\omega +\frac{T}{2}\dot{\omega }\right],0_{3}\right)\\ =\frac{1}{2}\left(-\frac{T^{2}}{4}\left[\omega \;\cdot \;\omega +T\omega \;\cdot \;\dot{\omega }+\frac{T^{2}}{4}\dot{\omega }\;\cdot \;\dot{\omega }\right],0_{3}\right)\\ =\frac{1}{2}\left(-\frac{T^{2}}{4}\left[\omega \;\cdot \;\omega \right]-\frac{T^{2}}{4}\left[T\omega \;\cdot \;\dot{\omega }+\frac{T^{2}}{4}\dot{\omega }\;\cdot \;\dot{\omega }\right],0_{3}\right)\\ =\frac{1}{2}\left[\left(-\frac{T^{2}}{4}\left[\omega \;\cdot \;\omega \right],0_{3}\right)+\left(-\frac{T^{2}}{4}\left[T\omega \;\cdot \;\dot{\omega }+\frac{T^{2}}{4}\dot{\omega }\;\cdot \;\dot{\omega }\right],0_{3}\right)\right]\\ =\frac{1}{2}\left[\frac{T^{2}}{4}\omega^{2}+\left(-\frac{T^{2}}{4}\left[T\omega \;\cdot \;\dot{\omega }+\frac{T^{2}}{4}\dot{\omega }\;\cdot \;\dot{\omega }\right],0_{3}\right)\right]\\ =\frac{T^{2}}{8}\omega^{2}+\underset{=v}{\underset{︸}{\left(-\frac{T^{3}}{8}\left[\omega \;\cdot \;\dot{\omega }+\frac{T}{4}\dot{\omega }\;\cdot \;\dot{\omega }\right],0_{3}\right)}} \end{array}$$

By insertion into Equation (B5), one gets:

(B7) $$exp\left(\frac{T}{2}\left(\omega +\frac{T}{2}\dot{\omega }\right)\right)=I_{q}+\frac{T}{2}\omega +\frac{T^{2}}{4}\dot{\omega }+\frac{T^{2}}{8}\omega^{2}+v+O\left(T^{3}\right)=I_{q}+\frac{T}{2}\omega +\frac{T^{2}}{8}\omega^{2}+\frac{T^{2}}{4}\dot{\omega }+O\left(T^{3}\right)$$

Note, that *v* is an error term of third order and is thus part of $O(T^{3})$. For further considerations, $q_{t+T}$ needs to be approximated. For this, the following identity is needed [60]:

(B8) $$\dot{q}=\frac{1}{2}\omega ⊙q$$

The time derivative is:

(B9) $$\begin{array}{c} \ddot{q}=\frac{1}{2}\dot{\omega }⊙q+\frac{1}{2}\omega ⊙\dot{q}\\ =\frac{1}{2}\dot{\omega }⊙q+\frac{1}{2}\omega ⊙\frac{1}{2}\omega ⊙q\\ =\left(\frac{1}{2}\dot{\omega }+\frac{1}{4}\omega^{2}\right)⊙q \end{array}$$

Now, the Taylor expansion of $q_{t+T}$ becomes:

(B10) $$\begin{array}{c} q_{t+T}=q_{t}+{\dot{q}}_{t}T+{\ddot{q}}_{t}\frac{T^{2}}{2}+O\left(T^{3}\right)\\ =q_{t}+\frac{T}{2}\omega_{t}⊙q_{t}+\frac{T^{2}}{2}\left(\frac{1}{2}{\dot{\omega }}_{t}+\frac{1}{4}\omega_{t}^{2}\right)⊙q_{t}+O\left(T^{3}\right)\\ =q_{t}+\frac{T}{2}\omega_{t}⊙q_{t}+\frac{T^{2}}{4}{\dot{\omega }}_{t}⊙q_{t}+\frac{T^{2}}{8}\omega_{t}^{2}⊙q_{t}+O\left(T^{3}\right)\\ =\left[I_{q}+\frac{T}{2}\omega_{t}+\frac{T^{2}}{4}{\dot{\omega }}_{t}+\frac{T^{2}}{8}\omega_{t}^{2}\right]⊙q_{t}+O\left(T^{3}\right)\\ =\left[\underset{=exp\left(\frac{T}{2}\left(\omega +\frac{T}{2}\dot{\omega }\right)\right)+O\left(T^{3}\right)}{\underset{︸}{I_{q}+\frac{T}{2}\omega_{t}+\frac{T^{2}}{8}\omega_{t}^{2}+\frac{T^{2}}{4}{\dot{\omega }}_{t}}}\right]⊙q_{t}+O\left(T^{3}\right) \end{array}$$

This means, Equation (B4) is a consistent approximation for $q_{t+T}$.  ☐

## Appendix C

This Appendix provides two terms (dynamic model, I2S calibration) in Equation (15), which were already proposed in [19]:

The motion of each IMU $I_{i}\in I$ from time step *t* to t+T, is modeled by taking the measured acceleration, $y_{i,t}^{a}$, and angular velocity, $y_{i,t}^{\omega }$, as control input [43], yielding $\forall I_{i}\in I$:

(C1a) $$\begin{array}{cc} I_{i,t+T}^{G} & =I_{i,t}^{G}+T{\dot{I}}_{i,t}^{G}+\frac{T^{2}}{2}R_{i,t}^{GI}\left(y_{i,t}^{a}-e_{i,t}^{\hat{p}}\right)+\frac{T^{2}}{2}g^{G} \end{array}$$

(C1b) $$\begin{array}{cc} {\dot{I}}_{i,t+T}^{G} & ={\dot{I}}_{i,t}^{G}+TR_{i,t}^{GI}\left(y_{i,t}^{a}-e_{i,t}^{\dot{\hat{p}}}\right)+Tg^{G} \end{array}$$

(C1c) $$\begin{array}{cc} q_{i,t+T}^{GI} & =q_{i,t}^{GI}⊙\exp\left(\frac{T}{2}\omega_{I,i,t}^{GI}+e_{i,t}^{\hat{q}}\right) \end{array}$$

Moreover, $\forall I_{i}\in I$:

(C2a) $$\begin{array}{cc} q_{i,t}^{GI} & =q_{i,t}^{GS}⊙q_{i,t}^{SI}⊙\exp\left(\frac{1}{2}e_{i,t}^{cq}\right) \end{array}$$

(C2b) $$\begin{array}{cc} I_{i,t}^{G} & =S_{i,t}^{G}+R_{i,t}^{GS}\left(I_{i,t}^{S}+e_{i,t}^{cp}\right) \end{array}$$

models the fact that IMU and segment poses are coupled through the I2S calibrations, up to some uncertainty that might compensate for soft tissue artifacts [19].

## Appendix D

This appendix summarizes the settings of all tuning parameters used in the evaluation in Section 3.

Table D1 provides the noise covariances used by all sensor fusion methods throughout all tests. Note, in order to provide comparable results, for the EKF-based methods, the measurement noise settings were kept equal and the process noise settings, due to the different motion models, where tuned per method. Tuning was done manually, first, on *sim-fast-artificial*, since this provided both slow and fast motion parts. The tuning parameters were then adapted based on *sim-slow* and *sim-fast*. The *Optitracker* did not require manual tuning for any parameter other than the magnetometer noise covariance. The latter was down-weighted, since this provided the best results on the above sequences. The same settings were then used for all test cases.

**Table D1:** Noise covariances used by all sensor fusion methods in all tests (cf. Table 1).

*Chaintracker*		*Quattracker segment|IMU*		*Optitracker*	
$\Sigma^{\ddot{\hat{\theta }}}$	3e+6	$\Sigma^{\hat{\omega }}$	$1e+4\;I_{3\times 3}$	$\Sigma^{cq}$	$I_{3\times 3}$
$\Sigma^{a}$	$0.8\;I_{3\times 3}$	$\Sigma^{\dot{\hat{\omega }}}$	$2e+4\;I_{3\times 3}$	$\Sigma^{cp}$	$I_{3\times 3}$
$\Sigma^{\omega }$	$0.03\;I_{3\times 3}$	$\Sigma^{\hat{p}}$	$3e+6\;I_{3\times 3}$	$\Sigma^{\hat{p}}$	$I_{3\times 3}$
$\Sigma^{m}$	0.01	$\Sigma^{a}$	$0.8\;I_{3\times 3}$	$\Sigma^{\dot{\hat{p}}}$	$I_{3\times 3}$
		$\Sigma^{\omega }$	$0.03\;I_{3\times 3}$	$\Sigma^{\hat{q}}$	$I_{3\times 3}$
		$\Sigma^{m}$	0.01	$\Sigma^{G}$	$I_{3\times 3}$
		$\Sigma^{p}$	$1e-7\;I_{3\times 3}$	$\Sigma^{m}$	$0.001\;I_{3\times 3}$
		$\Sigma^{G}$	$1e-7\;I_{3\times 3}$	$\Sigma^{q0}$	$I_{3\times 3}$
				$\Sigma^{p}$	$I_{3\times 3}$

For simulating realistic sensor noises, the following settings were used:

(D1) $$\begin{array}{c} y^{a}=y^{a}+e_{sim}^{a},\;y^{\omega }=y^{\omega }+e_{sim}^{\omega },\;y^{m}=y^{m}+e_{sim}^{m} \end{array}$$

with $\Sigma_{sim}^{a}=1.515\times 10^{-3}$, $\Sigma_{sim}^{\omega }=1.651\times 10^{-5}$ and $\Sigma_{sim}^{m}=0.01$.

The window size of the *Optitracker* was w=5 and the overlap was ov=1 throughout all tests.

## References

[1] Fong D.T.P., Chan Y.Y. (2010). The use of wearable inertial motion sensors in human lower limb biomechanics studies: A systematic review. Sensors.

[2] Patel S., Park H., Bonato P., Chan L., Rodgers M. (2012). A review of wearable sensors and systems with application in rehabilitation. J. NeuroEng. Rehabil..

[3] Hadjidj A., Souil M., Bouabdallah A., Challal Y., Owen H. (2013). Wireless sensor networks for rehabilitation applications: Challenges and opportunities. J. Netw. Comput. Appl..

[4] Zheng Y.L., Ding X.R., Poon C.C.Y., Lo B.P.L., Zhang H., Zhou X.L., Yang G.Z., Zhao N., Zhang Y.T. (2014). Unobtrusive sensing and wearable devices for health informatics. IEEE Trans. Biomed. Eng..

[5] Sabatini A. (2006). Quaternion-based extended Kalman filter for determining orientation by inertial and magnetic sensing. IEEE Trans. Biomed. Eng..

[6] Harada T., Mori T., Sato T. (2007). Development of a tiny orientation estimation device to operate under motion and magnetic disturbance. Int. J. Robot. Res..

[7] Young A.D., Ling M.J., Arvind D.K. Orient-2: A realtime wireless posture tracking system using local orientation estimation. Proceedings of the 4th Workshop on Embedded Networked Sensors (EmNets’07).

[8] Bergamini E., Ligorio G., Summa A., Vannozzi G., Cappozzo A., Sabatini A.M. (2014). Estimating orientation using magnetic and inertial sensors and different sensor fusion approaches: accuracy assessment in manual and locomotion tasks. Sensors.

[9] Ligorio G., Bergamini E., Pasciuto I., Vannozzi G., Cappozzo A., Sabatini A.M. (2016). Assessing the Performance of Sensor Fusion Methods: Application to Magnetic-Inertial-Based Human Body Tracking. Sensors.

[10] Ligorio G., Sabatini A.M. (2016). Dealing with Magnetic Disturbances in Human Motion Capture: A Survey of Techniques. Micromachines.

[11] Ligorio G., Sabatini A. (2015). A Novel Kalman Filter for Human Motion Tracking with an Inertial-based Dynamic Inclinometer. IEEE Trans. Biomed. Eng..

[12] Vignais N., Miezal M., Bleser G., Mura K., Gorecky D., Marin F. (2013). Innovative system for real-time ergonomic feedback in industrial manufacturing. Appl. Ergon..

[13] Bleser G., Damen D., Behera A., Hendeby G., Mura K., Miezal M., Gee A., Petersen N., Maçães G., Domingues H. (2015). Cognitive learning, monitoring and assistance of industrial workflows using egocentric sensor networks. PLoS ONE.

[14] Bleser G., Steffen D., Reiss A., Weber M., Hendeby G., Fradet L. (2015). Personalized Physical Activity Monitoring Using Wearable Sensors. Smart Health.

[15] Young A.D. Use of Body Model Constraints to Improve Accuracy of Inertial Motion Capture. Proceedings of the 2010 International Conference on Body Sensor Networks (BSN’10).

[16] Miezal M., Bleser G., Schmitz N., Stricker D. A generic approach to inertial tracking of arbitrary kinematic chains. Proceedings of the 8th International Conference on Body Area Networks.

[17] Roetenberg D., Luinge H., Slycke P. (2014). Xsens MVN: Full 6DOF Human Motion Tracking Using Miniature Inertial Sensors.

[18] Seel T., Schauer T., Raisch J. (2014). IMU-Based Joint Angle Measurement for Gait Analysis. Sensors.

[19] Kok M., Hol J., Schön T. An optimization-based approach to human body motion capture using inertial sensors. Proceedings of the 19th World Congress of the International Federation of Automatic Control (IFAC).

[20] El-Gohary M., McNames J. (2015). Human Joint Angle Estimation with Inertial Sensors and Validation with a Robot Arm. IEEE Trans. Biomed. Eng..

[21] Wagner J. (2004). Adapting the principle of integrated navigation systems to measuring the motion of rigid multibody systems. Multibody Syst. Dyn..

[22] Bouvier B., Duprey S., Claudon L., Dumas R., Savescu A. (2015). Upper Limb Kinematics Using Inertial and Magnetic Sensors: Comparison of Sensor-to-Segment Calibrations. Sensors.

[23] Cutti A.G., Giovanardi A., Rocchi L., Davalli A., Sacchetti R. (2008). Ambulatory measurement of shoulder and elbow kinematics through inertial and magnetic sensors. Med. Biol. Eng. Comput..

[24] Leardini A., Chiari L., Della Croce U., Cappozzo A. (2005). Human movement analysis using stereophotogrammetry: Part 3. Soft tissue artifact assessment and compensation. Gait Posture.

[25] Chen S., Brantley J., Kim T., Ridenour S., Lach J. (2013). Characterizing and Minimizing Sources of Error in Inertial Body Sensor Networks. Int. J. Autonom. Adapt. Commun. Syst..

[26] Shuster M.D. (1993). A survey of attitude representations. J. Astronaut. Sci..

[27] Wenk F., Frese U. Posture from motion. Proceedings of the International Conference on Intelligent Robots and Systems (IROS).

[28] Zhang Z.Q., Wu J.K. (2011). A Novel Hierarchical Information Fusion Method for Three-Dimensional Upper Limb Motion Estimation. IEEE Trans. Instrum. Meas..

[29] Jazwinski A.H. (2007). Stochastic Processes and Filtering Theory.

[30] Uhlmann J., Julier S., Durrant-Whyte H. (2000). A new method for the non linear transformation of means and covariances in filters and estimations. IEEE Trans. Autom. Control.

[31] Gustafsson F., Hendeby G. (2012). Some relations between extended and unscented Kalman filters. IEEE Trans. Signal Proc..

[32] Kok M., Pakazad S.K., Schön T.B., Hansson A., Hol J.D. A Scalable and Distributed Solution to the Inertial Motion Capture Problem. Proceedings of the 19th International Conference on Information Fusion.

[33] Skoglund M.A., Hendeby G., Axehill D. Extended Kalman filter modifications based on an optimization view point. Proceedings of the 18th International Conference on Information Fusion.

[34] Palermo E., Rossi S., Marini F., Patané F., Cappa P. (2014). Experimental evaluation of accuracy and repeatability of a novel body-to-sensor calibration procedure for inertial sensor-based gait analysis. Measurement.

[35] De Vries W., Veeger H., Cutti A., Baten C., van der Helm F. (2010). Functionally interpretable local coordinate systems for the upper extremity using inertial & magnetic measurement systems. J. Biomech..

[36] Favre J., Aissaoui R., Jolles B., de Guise J., Aminian K. (2009). Functional calibration procedure for 3D knee joint angle description using inertial sensors. J. Biomech..

[37] Seel T., Schauer T., Raisch J. Joint axis and position estimation from inertial measurement data by exploiting kinematic constraints. Proceedings of the International Conference on Control Applications (CCA).

[38] Bleser G., Hendeby G., Miezal M. Using Egocentric Vision to Achieve Robust Inertial Body Tracking under Magnetic Disturbances. Proceedings of the 10th International Symposium on Mixed and Augmented Reality (ISMAR-2011).

[39] Zatsiorsky V.M. (1998). Kinematics of Human Motion.

[40] Taetz B., Bleser G., Miezal M. Towards Self-Calibrating Inertial Body Motion Capture. Proceedings of the 19th International Conference on Information Fusion.

[41] Palermo E., Rossi S., Patanè F., Cappa P. (2014). Experimental evaluation of indoor magnetic distortion effects on gait analysis performed with wearable inertial sensors. Physiol. Meas..

[42] Denavit J., Hartenberg R.S. (1965). A Kinematic Notation for Lower-Pair Mechanisms Based on Matrices. J. Appl. Mech..

[43] Bleser G., Stricker D. (2009). Advanced tracking through efficient image processing and visual–inertial sensor fusion. Comput. Graph..

[44] Ungarala S., Dolence E., Li K. Constrained extended Kalman filter for nonlinear state estimation. Proceedings of the 8th International Symposium on Dynamics and Control of Process Systems.

[45] Miezal M., Taetz B., Schmitz N., Bleser G. Ambulatory inertial spinal tracking using constraints. Proceedings of the 9th International Conference on Body Area Networks.

[46] Black H.D. (1964). A passive system for determining the attitude of a satellite. AIAA J..

[47] Björck A. (1996). Numerical Methods for Least Squares Problems.

[48] Levenberg K. (1944). A Method for the Solution of Certain Non-Linear Problems in Least Squares. Q. Appl. Math..

[49] Thrun S., Burgard W., Fox D. (2005). Probabilistic Robotics (Intelligent Robotics and Autonomous Agents).

[50] Optitrack. http://www.optitrack.com/.

[51] Xsens. https://www.xsens.com/.

[52] Nyqvist H.E., Skoglund M.A., Hendeby G., Gustafsson F. Pose estimation using monocular vision and inertial sensors aided with ultra wide band. Proceedings of the International Conference on Indoor Positioning and Indoor Navigation (IPIN).

[53] Ligorio G., Sabatini A.M. (2015). A Simulation Environment for Benchmarking Sensor Fusion-Based Pose Estimators. Sensors.

[54] Tsai R., Lenz R. (1989). A new technique for fully autonomous and efficient 3D robotics hand/eye calibration. IEEE Trans. Robot. Autom..

[55] National Metrology Institute of Germany. http://www.ptb.de/en.

[56] De Vries W., Veeger H., Baten C., Van Der Helm F. (2009). Magnetic distortion in motion labs, implications for validating inertial magnetic sensors. Gait Posture.

[57] Angermann M., Frassl M., Doniec M., Julian B.J., Robertson P. Characterization of the indoor magnetic field for applications in localization and mapping. Proceedings of the International Conference on Indoor Positioning and Indoor Navigation (IPIN).

[58] Butterworth S. (1930). On the Theory of Filter Amplifiers. Wirel. Eng..

[59] Faber G.S., Chang C.C., Rizun P., Dennerlein J.T. (2013). A novel method for assessing the 3-D orientation accuracy of inertial/magnetic sensors. J. Biomech..

[60] Bleser G. (2009). Towards Visual-Inertial SLAM for Mobile Augmented Reality. Ph.D. Thesis.

